# YAP promotes osteogenesis and suppresses adipogenic differentiation by regulating β-catenin signaling

**DOI:** 10.1038/s41413-018-0018-7

**Published:** 2018-06-01

**Authors:** Jin-Xiu Pan, Lei Xiong, Kai Zhao, Peng Zeng, Bo Wang, Fu-Lei Tang, Dong Sun, Hao-han Guo, Xiao Yang, Shun Cui, Wen-Fang Xia, Lin Mei, Wen-Cheng Xiong

**Affiliations:** 1 Department of Neuroscience, Case Western Reserve University, Cleveland, OH 44106 USA; 2 Department of Neuroscience and Regenerative Medicine, Medical College of Georgia, Augusta University, Augusta, GA 30912 USA; 3 Louis Stokes Cleveland VAMC, Cleveland, OH USA; 4 Department of Rheumatology, Union Hospital, Tongji Medical College, Huazhong University of Science and Technology, 430072 Wuhan, Hubei China

## Abstract

YAP (yes-associated protein) is a transcriptional factor that is negatively regulated by Hippo pathway, a conserved pathway for the development and size control of multiple organs. The exact function of YAP in bone homeostasis remains controversial. Here we provide evidence for YAP’s function in promoting osteogenesis, suppressing adipogenesis, and thus maintaining bone homeostasis. YAP is selectively expressed in osteoblast (OB)-lineage cells. Conditionally knocking out *Yap* in the OB lineage in mice reduces cell proliferation and OB differentiation and increases adipocyte formation, resulting in a trabecular bone loss. Mechanistically, YAP interacts with β-catenin and is necessary for maintenance of nuclear β-catenin level and Wnt/β-catenin signaling. Expression of β-catenin in YAP-deficient BMSCs (bone marrow stromal cells) diminishes the osteogenesis deficit. These results thus identify YAP-β-catenin as an important pathway for osteogenesis during adult bone remodeling and uncover a mechanism underlying YAP regulation of bone homeostasis.

## Bone homeostasis: Genes play a balancing act

A key regulatory gene both promotes bone formation and suppresses production of fat-storing bone marrow cells, thus supporting the key process of bone remodeling. As adults, our bones are in a constant state of flux, constantly being dissolved and rebuilt to maintain a steady state known as homeostasis. An international team led by Wen-Cheng Xiong, of Case Western Reserve University, Cleveland, Ohio, studied the role of the gene ‘yes-associated protein’ (YAP), a transcription factor (gene controlling the activity of other genes). Using mice, they found that YAP is essential for osteogenesis, and prevents adipocyte (fat-storing cell) formation. Without a functional YAP gene, mice experienced a loss of a softer type of bone known as trabecular bone. The molecular pathways controlled by YAP may thus be important for bone remodeling in vertebrates.

## Introduction

YAP (yes-associated protein) is a transcriptional cofactor that is highly related to TAZ (transcriptional co-activator with PDZ binding motif). Both YAP and TAZ interact with TEA domain (TEAD) containing family transcriptional factors to induce gene transcription for diverse cellular processes, including cell proliferation and differentiation.^1–6^ Both YAP and TAZ are negatively regulated by the Hippo pathway, a conserved pathway that regulates organ size and tumorigenesis.^2,5,6^ Upon stimulation of the Hippo pathway, YAP is phosphorylated, which undergoes protein degradation or interaction with 14-3-3 for YAP cytoplasmic retention.^1–6^ When dephosphorylated, YAP enters nuclei and interacts with TEAD family transcriptional factors to induce gene expression.^1–6^ Recent studies indicate that, in addition to the Hippo pathway, YAP appears to be an integrator for cell proliferation and differentiation in response to various extracellular factors, including cell adhesion-driven mechanical cellular stress,^7^ bone morphogenetic proteins (BMPs),^1,8^ and Wnts.^4,9^ In addition to be a co-activator for TEAD family proteins, it serves as a co-regulator for other transcriptional factors that are crucial for bone homeostasis, such as phospho-Smad1/5/8,^8,10^ RUNX2,^11^ peroxisome proliferator-activated receptor-γ (PPARγ),^2^ signal transducer and activator of transcription factor 3 (STAT3),^12^ and β-catenin.^9^ Thus it is likely that YAP plays a role in bone homeostasis.

In this paper, we investigated YAP’s function in bone homeostasis in young adult mice. YAP is expressed in the osteoblast (OB) lineage, which includes committed OB precursors or progenitors, matrix-producing OBs, lining cells, and matrix-embedded osteocytes. By use of *Yap* conditional knockout (CKO) mice, Yap^Ocn-Cre^, we found that YAP is necessary to promote OB progenitor cell proliferation and differentiation, suppress mesenchymal stem cell's (MSC’s) adipogenic potential, and thus maintain trabecular bone (Tb) mass. We also showed that the OB-lineage YAP is required to maintain cytoplasmic and nuclear pools of β-catenin. Expression of β-catenin in *Yap*-deficient bone marrow-derived stromal cells (BMSCs) diminishes osteogenesis deficit. These results thus demonstrate YAP’s function in promoting osteogenesis and suppressing adipogenesis and reveal YAP’s positive regulatory role in β-catenin signaling during adult osteogenesis and bone homeostasis.

## Results

### Expression of YAP in OB-lineage cells

**Fig. 1:**
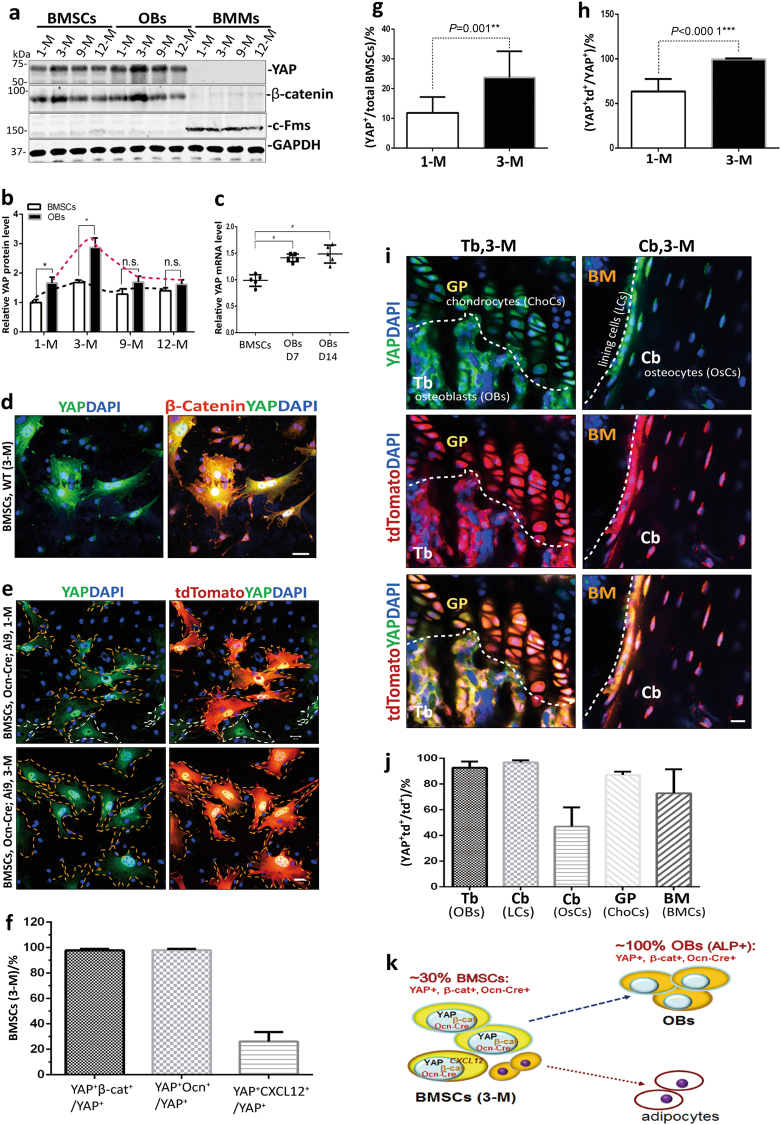
Expression of YAP in OB-lineage cells in culture and in vivo. **a**, **b** Western blot analysis of endogenous YAP levels in lysates of primary cultured BMSCs, OBs, and BMMs using the indicated antibodies (YAP, WH0010413M1, Sigma; β-catenin, Sigma, C7207). BMSCs and BMMs were derived from BM of mouse long bones at the indicated ages. OBs were in vitro differentiated from BMSCs at D14 cultures. The data were quantified by use of the NIH Image J software and presented in **b** (mean ± SD, *n* = 3 different cultures). *P < 0.05. **c** RT-PCR analysis of Yap gene expression in WT during OB differentiation. The data were present as mean ± SD (*n* = 5-different cultures). *P < 0.05. **d** Co-immunostaining analysis of YAP (1:200, mAb, WH0010413M1, Sigma) with β-catenin (1:2 000, pAb, C2206, Sigma) in BMSCs from WT mice (3-month old). **e** Immunostaining analysis of YAP (WH0010413M1, Sigma) in BMSCs from Ocn-Cre; Ai9 mice (1- and 3-month old). **f–h** Quantification analysis of data in **d**, **e**. The values of mean ± SD (*n* = 20) from three independent assays were presented. **P<0.01, ***P<0.000 1. **i**, **j** Immunohistochemical staining analysis of YAP/TAZ (pAb, #8418/D24E4, CST) in femur sections from 3-month-old Ocn-Cre;Ai9 mice. **k** Illustration of YAP expression in OB-lineage cells. In **a**–**h**, BMSCs were isolated from the indicated aged WT (**c**), Ocn-Cre; Ai9 (**e**–**h**), and CXCL12-dsRed mice (**f**). In **i**, **j**, the representative images are shown in **i**. The trabecular bone (Tb), cortical bone (Cb), growth plate (GP), and bone marrow (BM) are indicated. The YAP-tdTomato (Td) co-staining signals in OBs, osteocytes, lining cells, chondrocytes, and bone marrow cells were quantified [double-positive cells over total Td-positive cells in a selective region (%)] and presented in **j** (mean ± SD, *n* = 5 femur samples per genotype). *P < 0.05. Scale bar 20 µm.

To investigate YAP’s potential function in adult bone homeostasis, we first examined its expression in primary cultured bone cells, including BMSCs, OBs, and BMMs (bone marrow macrophages or monocytes), from various aged mice. Western blot analysis showed high levels of YAP in BMSCs and OBs, but little in BMMs (Fig. 1a), exhibiting a similar protein expression pattern as that of β-catenin (Fig. 1a), and implicating YAP’s expression in OB-lineage cells. The YAP expression in OBs derived from 1- to 3-month-old BMSCs were slightly higher than those in BMSCs (Fig. 1b, c), suggesting an age-dependency. YAP expression in BMSCs was further tested by co-immunostaining analysis of YAP and β-catenin. Notice that BMSCs were heterogeneous (Fig. 1d, e); fractions of BMSCs were positive for YAP (Fig. 1d, e); and nearly all (~100%) of YAP^+^ BMSCs had abundant β-catenin in their nuclei and cytoplasm (Fig. 1d, f). These results suggest that YAP is largely co-expressed with β-catenin in the OB-lineage cells.

To further test this view, we generated Ocn-Cre; Ai9 mice by crossing Ocn-Cre with Ai9 reporter mice (see Materials and methods) (Supplemental Fig. 1A) and examined whether YAP is co-expressed with Ocn-Cre-driven tdTomato (Td), a potential marker for the OB-lineage cells, in primary cultured BMSCs and OBs. As expected, the Td was positive in the OB-lineage cells, including fractions of BMSCs (Fig. 1e and Supplemental Fig. 1B) and OBs (Supplemental Fig. 1B-E), but negative in the BMMs (Supplemental Fig. 1B, C) and anti-perilipin-marked adipocytes (Supplemental Fig. 1F, G), supporting the view for Ocn-Cre to be largely expressed in the OB lineage.^13^ Remarkably, YAP was positive in nearly all of the Td^+^ BMSCs and OBs (Fig. 1e and Supplemental Fig. 1E), providing additional support for YAP’s selective expression in the OB lineage. Further characterization of those YAP and Td double-positive BMSCs revealed the following characteristics. First, the percentages of both YAP^+^ and Td^+^ BMSCs (over total BMSCs) were higher in 3-month-old mice than those in 1-month-old mice (Fig. 1g, h and Supplemental Fig. 1H). Second, both YAP^+^ and Td^+^ BMSCs appeared to be larger cells, with obvious larger nuclei in size than those in YAP or Td-negative BMSCs (Fig. 1e and data not shown). Third, Td^+^ or YAP^+^ BMSCs’ cell fate was toward OBs, but not toward adipocytes, upon in vitro differentiation under proper soluble factors (Fig. 1k and Supplemental Fig. 1). Nearly all (~100%) of the in vitro differentiated OBs were positive for YAP, Ocn-Cre-driven Td, and alkaline phosphatase (ALP) (an OB marker) (Supplemental Fig. 1D, E), but the Td^+^ or YAP^+^ adipocytes (marked by anti-perilipin) were nearly undetectable (Supplemental Fig. 1F, G).

We then asked whether YAP is co-distributed with Td in femur bone sections from 3-month-old Ocn-Cre; Ai9 mice. Indeed, immunofluorescence staining analysis showed that nearly all of YAP^+^ cells were co-positive for Td (Fig. 1i, j); and these YAP and Td double-positive cells appeared to be OB-lineage cells, including osteoblastic-like cells in Tb, lining cells, and osteocytes in cortical bone (Cb) regions (Fig. 1i, j and Supplemental Fig. 2). Together, these results provide in vitro and in vivo evidence for YAP to be co-expressed with Ocn-Cre in the OB-lineage cells.

In addition to the OB-lineage, both YAP- and Ocn-Cre-positive signals were detected in portions of chondrocytes at the growth plate (GP) (Fig. 1i, j and Supplemental Fig. 2) and in fractions of C-X-C chemokine motif ligand 12 (CXCL12)-marked CAR (CXCL12-abundant reticular) cells and NG2-labeled pericytes (Fig. 1f and data not shown), in line with a report for Ocn-Cre to be partially expressed in non-OB-lineage cells in bone marrows in vivo.^14^

### Trabecular bone loss in *Yap* conditionally knocking out mice, Yap^Ocn-Cre^

YAP’s expression in the OB-lineage implicates its function in osteogenesis. To test this view, we generated Yap^Ocn-Cre^ mice by crossing Yap^f/f^ with Ocn-Cre. YAP (~70 kDa) was markedly reduced in Yap^Ocn-Cre^-BMSCs and OBs, compared with controls (Supplemental Fig. 3A-C), demonstrating YAP antibody specificity and confirming Yap^Ocn-Cre^ mouse identity. However, a smaller molecular weight protein (~50 kDa) was detected by the anti-Yap antibody (longer exposure), which might be due to its cross-reactivity to YAP homolog, TAZ, because this 50 kDa protein was not reduced in Yap^Ocn-Cre^ BMSCs and recognized by anti-TAZ antibody (Supplemental Fig. 3A, B).

**Fig. 2:**
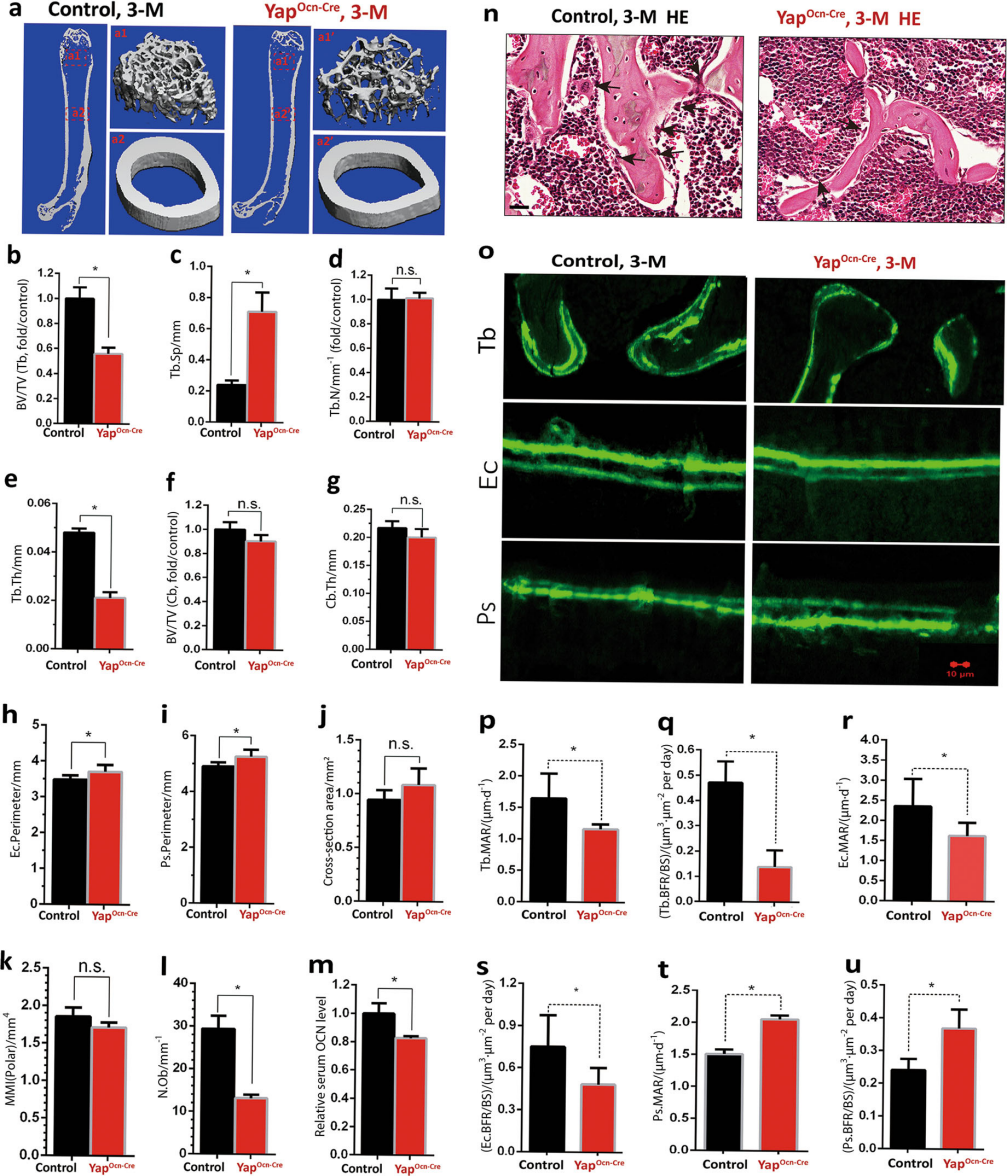
Trabecular bone loss and decreases of bone formation in Yap^Ocn-Cre^ mice. **a**–**k** µCT analysis of femurs from 3-month-old Yap^Ocn-Cre^ and control (ctrl) (Yap^f/f^) littermates. Five different male mice of each genotype were examined blindly. Representative images are shown in **a**. The 3D images shown on the right (a1, a1′, a2, and a2′) were derived from the marked corresponding regions of the femurs in the left images. Quantification analyses are presented in **b**–**k**. Note that the trabecular bone (Tb) volumes over total volumes (BV/TV), trabecular separation (Tb.Sp), and trabecular thickness (Tb.Th) but not trabecular numbers (Tb.N) by direct model of µCT analysis were all deficient in Yap^Ocn-Cre^ as compared with the ctrls. The cortical BVs over TVs (BV/TV), cortical thickness (Cb.Th), and cortical cross-section area and polar mean moment of inertia (MMI, Polar) were unchanged in Yap^Ocn-Cre^ mice (**f**, **g**, **j**, **k**). Note that endocortical perimeter (Ec.Perimeter) and periosteal perimeter (Ps.Perimeter) were increased (**h**, **i**). The data shown in **b**–**k** are: Means ± SD, 5 bone samples/each genotype, *P < 0.05. **l** Bar graph showing the number of OBs/unit bone surface (1 mm) in 3-month-old Yap^Ocn-Cre^ and ctrl (Yap^f/f^) femur sections, representative images of H&E staining analyses of OBs in femurs from 3-month-old Yap^Ocn-Cre^ and ctrl (Yap^f/f^) littermates in (**n**), Scale bars, 20 µm, arrows indicate OBs. **m** Reduced serum levels of osteocalcin, measured by ELISA assays, in 3-month-old Yap^Ocn-Cre^ mice (mean ± SD, *n* = 5, male mice). **o**–**u** Attenuated bone formation, detected by dynamic histomorphometric measurements of double fluorescent-labeled femurs, in 3-month-old Yap^Ocn-Cre^ mice. Ctrl and Yap^Ocn-Cre^ mice (male) at the age of P76 were injected (intraperitoneal) with fluorochrome-labeled calcein green (10 mg·kg^-1^, Sigma–Aldrich), and 12 days after, they were re-injected to label active bone-forming surfaces. Two days after the second injection, mice were sacrificed and their femurs were fixed, sectioned, and viewed by fluorescence microscope (**o**). Scale bar, 10 µm. The trabecular (Tb), endocortical (Ec), and periosteum (Ps) mineral apposition rate (MAR) (calculated in µm·day^-1^) (**p**, **r**, **t**), and their bone-formation rate (BFR = MAR×minerization surface/bone surface) (**q**, **s**, **u**) are illustrated. In **o**–**u**, the values of mean ± SD from 5 different male mice per genotype are shown. *P < 0.05.

Yap^Ocn-Cre^ mice displayed normal growth with comparable body weight to that of control littermates (Yap^f/f^) (Supplemental Fig. 3D, E). We then examined their long bone (femur) mass (at age of 3-month old) by microCT (µCT) analysis, as the Ocn-Cre activity is more active at this age. As shown in Fig. 2a, b, Tb volumes over total volumes were markedly reduced in Yap^Ocn-Cre^ mice, compared with that of littermate controls. In agreement, the trabecular space (Tb.Sp) but not trabecular numbers (Tb.N), were increased in Yap^Ocn-Cre^ mice, and trabecular thickness (Tb.Th) was decreased in Yap^Ocn-Cre^ mice (Fig. 2c–e). However, the cortical bone volumes (BV), cortical bone thickness (Cb.Th), cross-section area, and polar mean moment of inertia were unchanged (Fig. 2f, g, j, k). It is of interest to note that the endocortical (Ec.) and peristeal (Ps.) perimeter were increased in Yap^Ocn-Cre^ mice (Fig. 2h, i). The number of OBs/unit bone surface was reduced in Yap^Ocn-Cre^ mice by hematoxylin and eosin (H&E) staining (Fig. 2l, n). Similar deficits (Tb loss, decreased OB number, increased perimeter, and normal cortical bone volumes) were obtained in Yap^Ocn-Cre^ female mice (Supplemental Fig. 4). These results thus demonstrate a Tb loss in Yap^Ocn-Cre^ mice, indicating YAP’s function in maintaining adult Tb homeostasis.

### Decreased bone formation, but normal bone resorption, in Yap^Ocn-Cre^ mice

To understand how YAP regulates Tb homeostasis, OB-mediated bone formation and osteoclast (OC)-mediated bone resorption, both processes essential for bone homeostasis, were examined. The bone resorption was first examined by measuring serum levels of pyridinoline, a marker associated with bone turnover. No significant difference was detected between control (Yap^f/f^) and Yap^Ocn-Cre^ mice (Supplemental Fig. 5A), suggesting little, if there is any, role of YAP in this process. This view was further supported by the observation that TRAP^+^ OC number per unit bone surface area of Yap^Ocn-Cre^ mice were comparable to that of their controls (both male and female mice) (Supplemental Fig. 5B-E). Little difference was observed in TRAP^+^ multi-nuclei cells (MNCs) by receptor activator of NF-κB ligand (RANKL)-induced in vitro OC differentiation of BMMs from control and Yap^Ocn-Cre^ mice (Supplemental Fig. 5F, G). However, there were slightly more TRAP^+^ MNCs formed in the in vitro OC differentiation assay when wild-type (WT)-BMMs were co-cultured with OBs from Yap^Ocn-Cre^ mice than that with control OBs (Supplemental Fig. 5H, I). Together, these results indicate that YAP functions within Ocn-cre-positive OB-lineage cells, and it plays a little role in regulating osteoclastogenesis in vitro and in vivo.

We next examined bone formation by carrying out the following two assays. First, measuring serum levels of osteocalcin, a maker of bone formation, by enzyme-linked immunosorbent assay revealed a significant reduction in Yap^Ocn-Cre^ mice, compared with that of littermate controls (Fig. 2m), suggesting a decrease in bone formation. Second, dynamic measurements of non-decalcified femur and tibia bone sections, which were double labeled by two injections of fluorescent calcein (at 12-day interval) in control and Yap^Ocn-Cre^ mice, demonstrated reductions in Tb.MAR (trabecular mineral apposition rate), Tb.BFR (Tb formation rate), Ec.MAR (endocortical MAR), and Ec.BFR (endocortical BFR) in 3- but not 1-month-old Yap-CKO mice, as compared with that of littermate controls (Fig. 2o–s, data not shown). Notice that the Ps.MAR (periosteal MAR) and Ps.BFR (periosteal BFR) were increased in Yap^Ocn-Cre^ mice (Fig. 2o, t, u). Taken together, these results, in line with µCT analysis, revealed a positive role for YAP in bone formation and suggest that the reduced bone formation may contribute to the Tb loss in Yap^Ocn-Cre^ mice.

### Impaired OB-differentiation, but increased adipocyte formation, in Yap^Ocn-Cre^ BMSC cultures

**Fig. 3:**
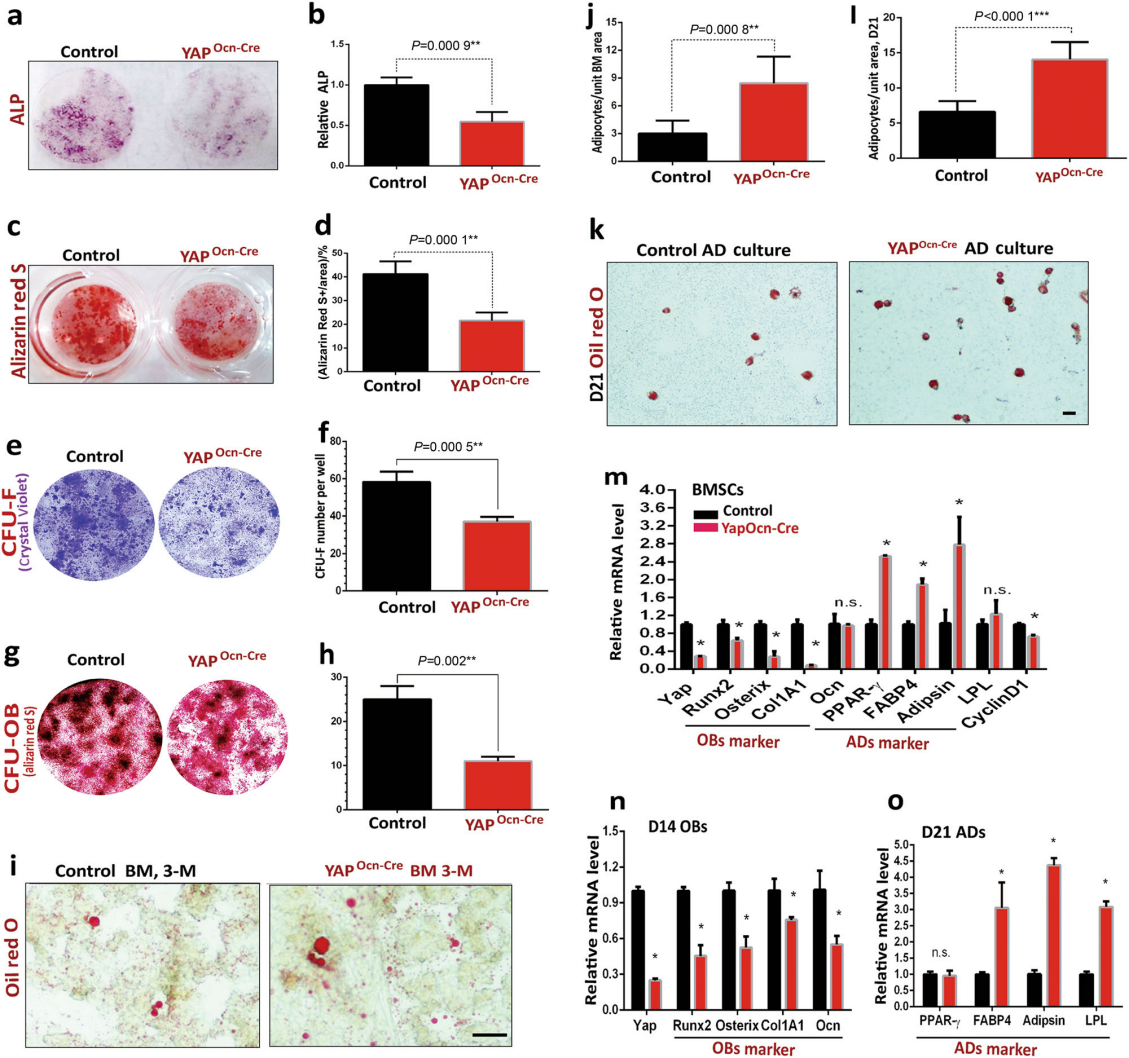
Reductions in OB differentiation and function and increases in adipocyte differentiation in Yap^Ocn-Cre^-BMSC/OB cultures. **a**, **b** In vitro OB differentiation was decreased in Yap^Ocn-Cre^-BMSC cultures. ALP staining images at D14 cultures are shown in **a**, and ALP activity (ALP-positive area/over total area, normalized by three ctrl cultures) is presented in **b**. **c**, **d** In vitro OB function (viewed by Alizarin Red staining) was attenuated in Yap^Ocn-Cre^-OB cultures. At D28 of BMSC/OB cultures, cells were stained for Alizarin Red S (**c**), and the data were quantified and illustrated in **d**. In **b**, **d**, the values of mean ± SD (*n* = 3 different cultures) are presented.  **P<0.01. **e**–**h** Both CFU-F and CFU-OB were reduced in Yap^Ocn-Cre^-BMSC cultures. CFU-F and CFU-OB assays are described in the supplemental methods. The Crystal Violet (**e**) and Alizarin Red S (**g**) staining images and their quantifications (mean ± SD, *n* = 3 different cultures) are shown. **i**, **j** Oil Red O staining analysis showed an increase in bone marrow fat-containing cells in Yap^Ocn-Cre^ mice (3-month old). **i**, representative images and **j**, quantification analysis of bone marrow adipocytes over total bone marrow cells in femur mid diaphysis. (mean ± SD, *n* = 5 mice/genotype, male). **P<0.01. **k**, **l** BMSCs from ctrl (Yap^f/f^) and Yap^Ocn-Cre^ mice (3-month old) were induced for adipocyte (AD) differentiation, and at D21 cultures, they were stained with Oil Red O. **k**, adipocyte images; and **l**, quantification analysis (mean ± SD, *n* = 3 different experiments). Scale bar, 100 µm, ***P<0.000 1. **m**–**o** Real-time PCR analyses of the indicated genes’ expression in ctrl and Yap^Ocn-Cre^ BMSCs, OBs (D14), and ADs (D21) (mean ± SD, *n* = 3 different assays). *P < 0.05.

To investigate cellular mechanisms underlying YAP regulation of bone formation, we asked whether YAP is necessary for in vitro OB differentiation from primary cultured BMSCs. OB differentiation, viewed by ALP enzymatic activity staining, was lower in cultures of Yap^Ocn-Cre^ than that of controls (Fig. 3a, b). Also decreased were calcified bone matrix stained by Alizarin Red S staining in Yap^Ocn-Cre^ cultures (Fig. 3c, d). In addition, both colony-forming unit fibroblast (CFU-F) and CFU-OBs were reduced in Yap^Ocn-Cre^ cultures (Fig. 3e–h). These results suggest that YAP in Ocn-Cre^+^ cells is required to promote OB-proliferation, differentiation, and function.

In addition to the Tb loss, Yap-CKO femurs contained many large oval-shaped vacuoles, likely to be marrow adipocytes, by H&E staining analysis (male and female) (Supplemental Fig. 6A-D). We further tested this view by Oil Red O staining analysis, which marks bone marrow fat or adipocytes. Indeed, more Oil Red O stained adipocytes were detected in Yap^Ocn-Cre^ bone marrows than that of controls (Fig. 3i, j). We then examined adipocyte differentiation from BMSCs in culture. Adipocytes or CFU-adipocytes viewed by Oil Red O staining or anti-perilipin immunostaining were higher in Yap^Ocn-Cre^ cultures than that of controls (Fig. 3k, l and Supplemental Fig. 6E-H), in line with the in vivo observations. Furthermore, we compared gene expression profiles between control and Yap-CKO BMSCs, OBs, and adipocytes. The mRNAs of genes critical for OB genesis, such as Runx2, osterix, and Col1A1, as well as cyclin D1 (a cell cycle marker), were largely reduced in Yap^Ocn-Cre^ BMSCs/OBs; however, the expression levels of genes for adipocyte formation (e.g., PPARγ, FABP4, and adipsin) were elevated in Yap^Ocn-Cre^ BMSCs/adipocytes (Fig. 3m–o). Together, these results demonstrate that YAP in Ocn-Cre^+^ cells not only promotes OB genesis but also suppresses adipocyte formation, likely due to YAP’s transcriptional regulation of relevant gene expression.

### Reduced cell proliferation in Yap^Ocn-Cre^ BMSC and OB cultures and in vivo

**Fig. 4:**
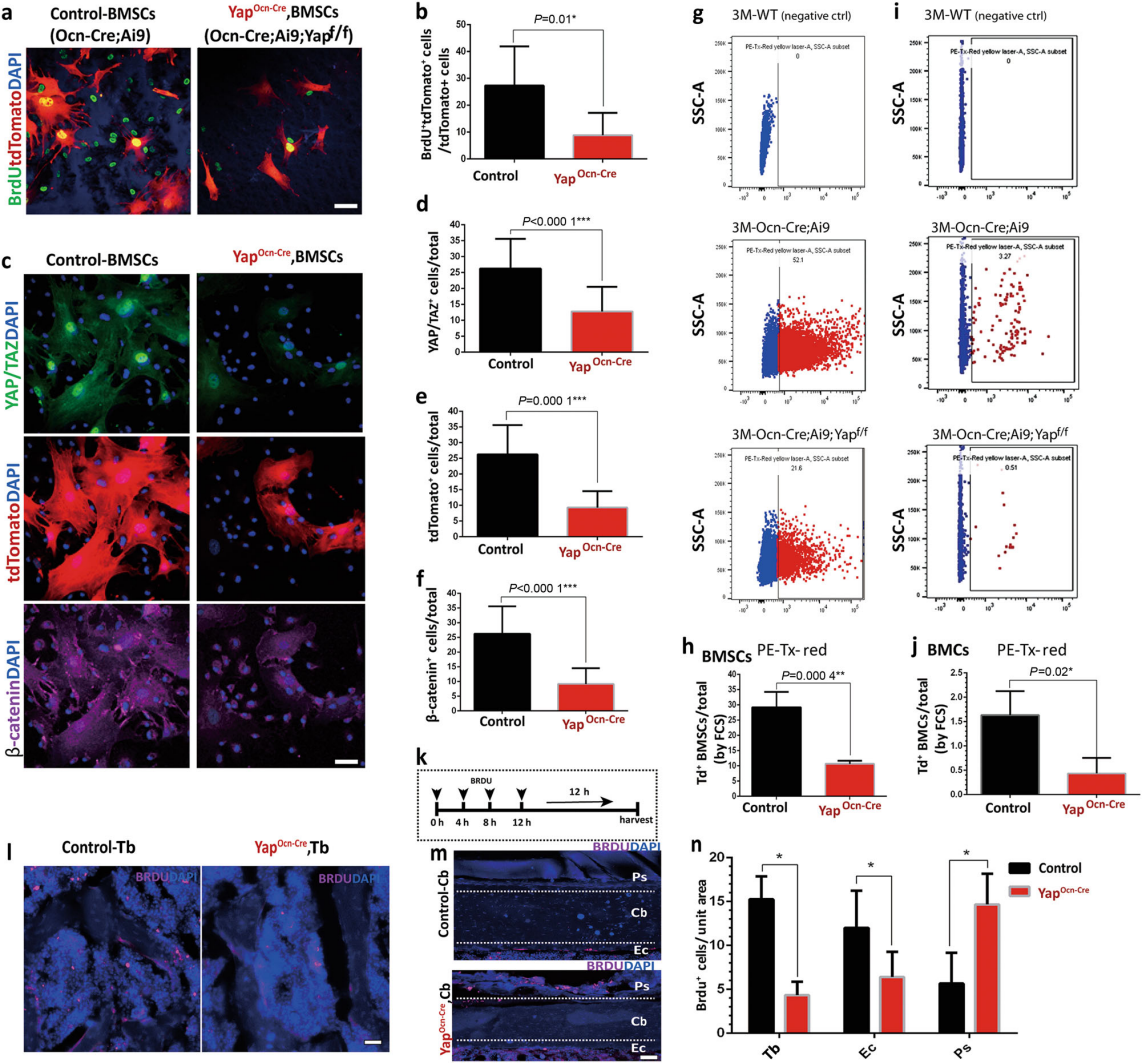
Declines of cell proliferation and OB-progenitor cells in Yap^Ocn-Cre^ mice. **a**, **b** Reductions in OB-progenitor cell proliferation in Yap^Ocn-Cre^ BMSC culture. BMSCs were incubated with BrdU (10 μmol·L^-1^, 2 h) and then subjected to anti-BrdU immunostaining analysis. **a**, representative images, Scale bar, 20 µm. **b**, BrdU^+^ Td^+^ cells over total Td^+^ cells (mean ± SD, *n* = 50 from 3 different cultures), *P<0.05. **c**–**f** Decreases in Td and β-catenin double-positive BMSCs from Yap^Ocn-Cre^ mice. BMSCs from 3-month-old ctrl (Ocn-Cre;Ai9) and Yap-CKO (Ocn-Cre;Ail9;Yap^f/f^) mice were subjected to co-immunostaining analyses using the indicated antibodies (YAP, WH0010413M1, Sigma; β-catenin, Sigma, C2206). **c**, representative images, Scale bar, 20 µm. **d**–**f**, quantification analyses (mean ± SD, *n* = 50 from 3 different cultures), ***P<0.000 1. **g**–**j** FACS analysis of Td^+^ cells in cultured BMSCs (**g**, **h**) and isolated total bone marrow cells (BMCs) (**I**, **j**) from 3-month-old WT, Ocn-Cre;Ai9, and Ocn-Cre;Ai9;Yap^f/f^ mice. Representative FACS data were shown in **g**, **i**. The quantification analyses were presented in **h**, **j**. (Mean ± SD, *n* = 3 from 3 different cultures or mice. **P < 0.01). **k**–**n** Analysis of BrdU^+^ proliferative cells in ctrl and Yap^Ocn-Cre^ femur bone sections. BrdU (100 μg·kg^-1^) was injected (i.p. four times over a 12 h period at 0, 4, 8, 12 h) into ctrl and Yap^Ocn-Cre^ mice (3 months), and 12 h after the last injection, mice were sacrificed (**k**), and their femur bone sections were subjected to anti-BrdU antibody immunostaining analysis. **l**–**m**, representative images; **n**, quantification analysis of BrdU^+^ cell density in Tb (trabecular bone), Ec (endocortical bone surface), and Ps (periosteum) (Mean ± SD, *n* = 3 male mice. *P < 0.05).

YAP is known to be critical for cell proliferation in various cell types.^2,3,6,8^ We thus asked whether YAP regulates OB-progenitor cell proliferation, a critical event for osteogenesis. In line with this view were observations of decreased number of OBs/unit bone surface in Yap^Ocn-Cre^ mice (Fig. 2l, n) and lower CFU-F in Yap^Ocn-Cre^ cultures (Fig. 3e, f). To further test this view, we examined bromodeoxyuridine (BrdU)-marked cell proliferation in control (Ocn-Cre; Ai9) and Yap-CKO (Ocn-Cre; Ai9; Yap^f/f^) BMSC cultures. OB progenitors in BMSC cultures could be marked by Ocn-Cre-driven Td. BMSCs from control and Yap-CKO mice were incubated with BrdU (10 μmol·L^-1^ for 2 h), which marks dividing cells, and then subjected to immunostaining analysis with anti-BrdU. Indeed, BrdU^+^ BMSCs over total BMSCs were lower in Yap-CKO cultures, compared with that of controls (Fig. 4a). A decrease in BrdU and Td double-positive cells over total Td^+^ cells was detected in Yap-CKO cultures (Fig. 4a, b), indicating a reduction of cell proliferation in Yap-CKO OB progenitors and providing a cellular mechanism underlying YAP regulation of osteogenesis. In agreement, Td^+^ BMSCs over total BMSCs were reduced in the Yap-CKO cultures (Fig. 4c–e): ~25% of Td^+^ BMSCs over total BMSCs from 3-month-old control mice were detected; however, only ~10% of these cells were detected in Yap-CKO cultures (Fig. 4c–e). β-Catenin^+^ BMSCs over total BMSCs were also decreased in Yap-CKO cultures (Fig. 4c, f). The reductions of Td^+^ cells in Yap^Ocn-Cre^ BMSC and BMC (bone marrow cell) cultures were further confirmed by fluorescence-activated cell sorting analysis (Fig. 4g–j). Finally, we examined femur bone cell proliferation in control and Yap^Ocn-Cre^ mice injected with BrdU (100 mg·kg^-1^, intraperitoneally four times over a 12 h period at 0, 4, 8, and 12 h and sacrificed 12 h after the last injection) (Fig. 4k). BrdU-labeled cells were lower in the Tbs and endocortical bone surface of Yap^Ocn-Cre^ mice than those of controls (Fig. 4l–n). However, more BrdU^+^ cells in the periosteal region of the cortical bones were detected in the Yap^Ocn-Cre^ mice (Fig. 4m, n). These results, in line with observations from the bone-formation assay, provide additional support for YAP to promote OB-progenitor proliferation, which may underlie YAP regulation of osteogenesis.

### YAP stabilization of β-catenin protein in BMSC, BM-OB, and MC3T3 cells

To understand how YAP regulates osteogenesis, we compared protein levels of β-catenin and phospho-Smad1/5/8 (pSmad1/5/8) between control and Yap^Ocn-Cre^ OBs, because both signaling proteins are crucial for OB genesis^15,16^ and regulated by YAP in various cell types.^8–10^ Western blot analysis showed a comparable level of pSmad1/5/8 between Yap^Ocn-Cre^ and control OBs but much lower level of β-catenin in Yap^Ocn-Cre^-BM-OBs (Supplemental Fig. 7A, B). The reduction in β-catenin was detected not only in total lysates of Yap^Ocn-Cre^ BM-OBs but also in their cytoplasm and nuclei fractions, as compared with that of control-BM-OBs (Supplemental Fig. 7C, D). Both total and active β-catenin, but not the Wnt inhibitor DKK1, were reduced in Yap^Ocn-Cre^-BMSCs and OBs (Supplemental Fig. 7E-H), suggesting a reduction of β-catenin activity in Yap^Ocn-Cre^ OB-lineage cells. This view was further supported by observations of decreased expression of Wnt target genes (e.g., RUNX2, OPG, RANKL, Cyclin D1),^17–19^ but not the Wnt inhibitor DKK1, in Yap-CKO BMSCs (Supplemental Fig. 7I).

**Fig. 5:**
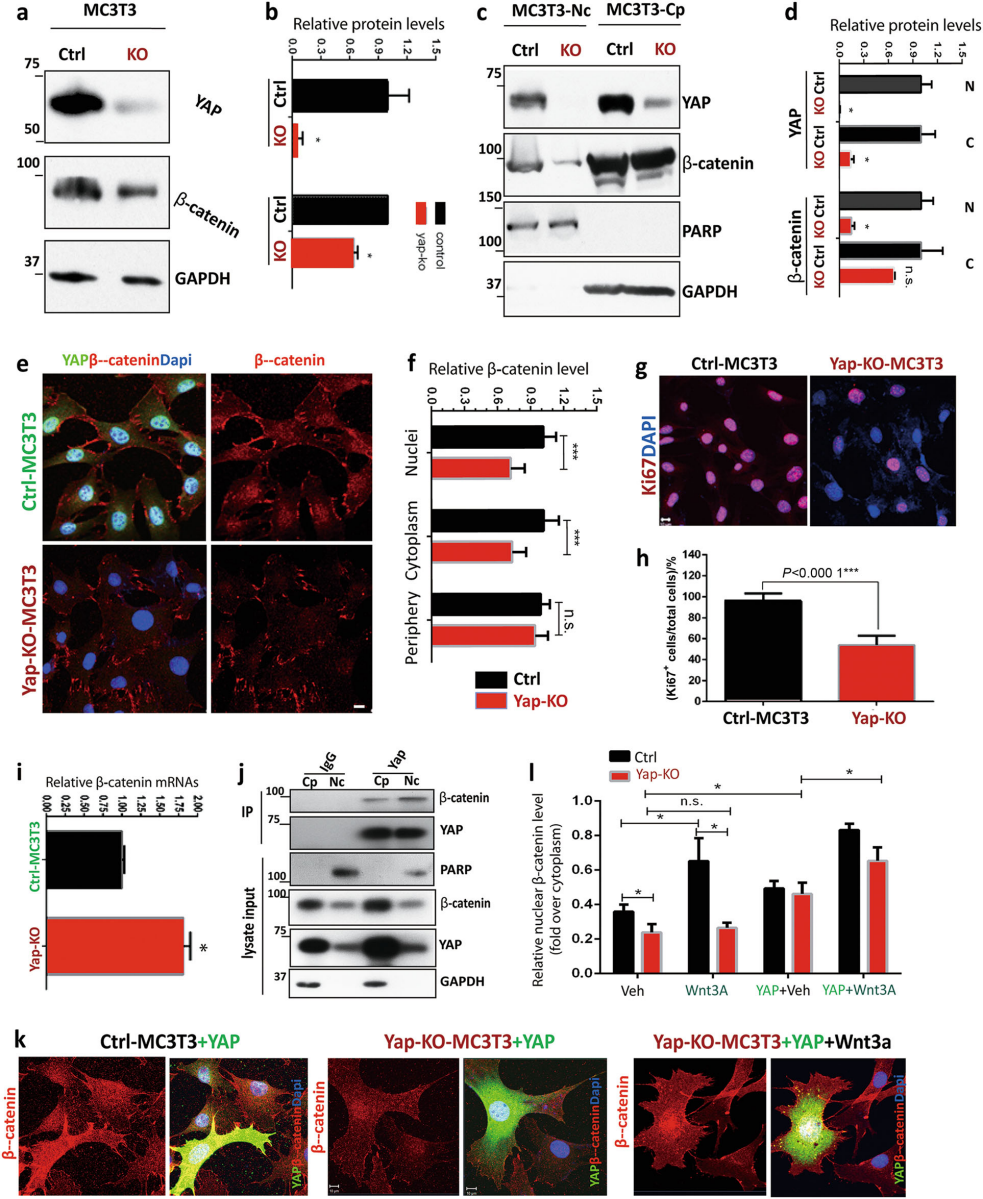
β-Catenin falling off in YAP-KO MC3T3 cells. **a**–**d** Western blot analysis of YAP and β-catenin (C7207, Sigma) in ctrl and Yap-KO MC3T3 cell lines. Yap-KO MC3T3 cell line is generated by CRISPR-Cas9 strategy. Total lysates (**a**, **b**) and lysates of nuclei and cytoplasmic fractions (**c**, **d**) of ctrl and Yap-KO MC3T3 cells were subjected to immunoblot analysis using the indicated antibodies. **a**, **c**, representative blots; **b**, **d**, quantification analysis (mean ± SD, *n* = 3-different cultures, *P < 0.05). **e**, **f** Co-immunostaining analysis of β-catenin (C2206, Sigma) and YAP in ctrl and Yap-KO MC3T3 cells. **e**, representative images. Scale bar, 10 µm. **f**, quantification data (mean ± SD, *n* = 50 from 3 different cultures). ***P < 0.000 1. **g**, **h** Reduced cell proliferation in Yap-KO MC3T3 cells revealed by immunostaining analysis of Ki67 (a marker for cell proliferation). **g**, representative images, Scale bar, 20 µm. **h**, quantification data (mean ± SD, *n* = 50 from 3 different cultures). ***P < 0.000 1. **i** Real-time PCR analysis of β-catenin mRNAs in ctrl and Yap-KO MC3T3 cells (mean ± SD, *n* = 3). **j** Co-immunoprecipitation analysis. The nuclear and cytoplasmic MC3T3 cell lysates were immunoprecipitated with anti-Yap (WH0010413M1, Sigma) and non-specific (nS) IgG. The resulting precipitates were subjected to immunoblot analysis using the indicated antibodies. Input, ~50 µg lysates. **k**, **l** Co-immunostaining analysis of β-catenin (C2206, Sigma) and YAP in ctrl and Yap-KO MC3T3 cells expressing YAP-GFP in the presence or absence of Wnt3A. **k**, representative images, Scale bar, 10 µm. **l**, quantification analysis (β-catenin fluorescence intensity in nuclei over cytoplasm by use of the NIH Image J software) (mean ± SD, *n* = 20 cells from 3 different experiments).

The reductions in putative OB-progenitor cells (marked by Ocn-Cre and β-catenin) in Yap^Ocn-Cre^ BMs raise a concern whether the decreased β-catenin in BMSCs/BM-OBs could be a consequence of the phenotype (an indirect event) and/or due to a direct regulation of β-catenin level by YAP. To address this concern, we examined YAP’s effect on β-catenin in MC3T3 cells, an OB-cell line, to prevent YAP’s in vivo or indirect effect on OB progenitors. YAP gene was knocked out (KO) in MC3T3 cells by use of the CRISPR-Cas9 strategy (see Supplemental Methods). When YAP was depleted, β-catenin levels in total lysates or lysates of nuclear fractions of MC3T3 cells were significantly lower than those of control cells (Fig. 5a–d). β-Catenin reduction was reconfirmed by immunostaining analyses (Fig. 5e, f). A similar β-catenin decrease was also detected in MC3T3 cells that were transiently transfected with shRNA-Yap (Supplemental Fig. 8). In addition, Ki67-marked proliferative cells were significantly decreased in Yap-KO MC3T3 cells (Fig. 5g, h). These results, in agreement with the results obtained in Yap-CKO BMSCs/BM-OBs, support the view for YAP to promote OB cell proliferation and maintain the nuclear level of β-catenin.

To understand how YAP regulates β-catenin levels, we first examined β-catenin’s mRNA levels in control and Yap-KO MC3T3 cells, as YAP is a transcriptional factor. To our surprise, a slight increase in β-catenin’s mRNAs in Yap-KO cells was revealed by real-time PCR analysis (Fig. 5i), excluding a transcriptional mechanism underlying the decrease of β-catenin. We then asked whether YAP forms a complex with β-catenin, thus stabilizing β-catenin protein levels. Co-immunoprecipitation analysis showed that β-catenin was detected in the anti-Yap, but not the non-specific immunoglobulins, immuneprecipitates from lysates of both nuclear and cytoplasmic fractions (Fig. 5j); however, more Yap-associated β-catenin was detected in the lysates of nuclear fractions than that of cytoplasmic fractions (Fig. 5j). To further test whether YAP stabilize β-catenin levels, YAP expression plasmid was transiently transfected into Yap-KO MC3T3 cells. β-Catenin levels were markedly elevated in both control and Yap-KO MC3T3 cells expressing YAP, compared with those of untransfected cells (Fig. 5k, l). Additionally, Wnt3A treatment increased nuclear β-catenin in control but not in Yap-KO MC3T3 cells (Fig. 5k, l). These results thus support the view for YAP interacting with and stabilizing β-catenin protein.

### YAP regulation of β-catenin and its signaling in vivo

We then asked whether YAP regulates β-catenin and its signaling in vivo. β-Catenin level was first examined by immunostaining analysis of femur bone sections of control (Ocn-Cre; Ai9) and Yap CKO (Yap^f/f^; Ocn-Cre; Ai9) mice. Both Td- and β-catenin-positive OB-lineage cells were markedly reduced in Yap-CKO mice compared with that of controls (Supplemental Fig. 9A-D), supporting the view for a decreased OB genesis in vivo. In the remaining Td-positive OBs and osteocytes, β-catenin was nearly undetectable in Yap-CKO mice (Supplemental Fig. 9A-D), in line with the results from BMSC/OB/MC3T3 cultures, providing evidence for osteoblastic YAP to maintain β-catenin level in vivo. Note that, whereas β-catenin was reduced in osteocytes of Yap-CKO cortical bones, Td^+^ osteocytes remained comparable to that of controls (Supplemental Fig. 9B, D), in agreement with the result of unchanged cortical bone volumes. Although total Td^+^ osteocytes in the Yap-CKO cortical bones were similar to that of controls, careful analysis of the distribution of the Td^+^ osteocytes showed a reduction in the endocortical regions but a slight increase in the region toward periosteum (Supplemental Fig. 9E, F), in line with the result of BrdU-labeling proliferation assay (Fig. 4m, n) and implicating a compensatory effect that may occur in this region of Yap-CKO cortical bones.

We next examined whether β-catenin signaling is altered in Yap CKO mice. The Axin2-LacZ reporter mice, whose expression depends on β-catenin signaling in the nuclei,^20^ were crossed with Yap^Ocn-Cre^ to obtain Yap^Ocn-Cre^; Axin2-LacZ mice. In comparison with the control mice (Axin2-LacZ), the LacZ signals in Yap-CKO mice, likely to be OB-lineage cells in both trabecular and cortical bone regions, were reduced (Supplemental Fig. 10). This view was further supported by observations of decreased active β-catenin in both trabecular and cortical bone regions in YAP CKO mice (Supplemental Fig. 11), suggesting an important role of YAP in regulating β-catenin signaling in vivo.

### Restore of osteogenesis from Yap^Ocn-Cre^-BMSCs by expression of β-catenin

**Fig. 6:**
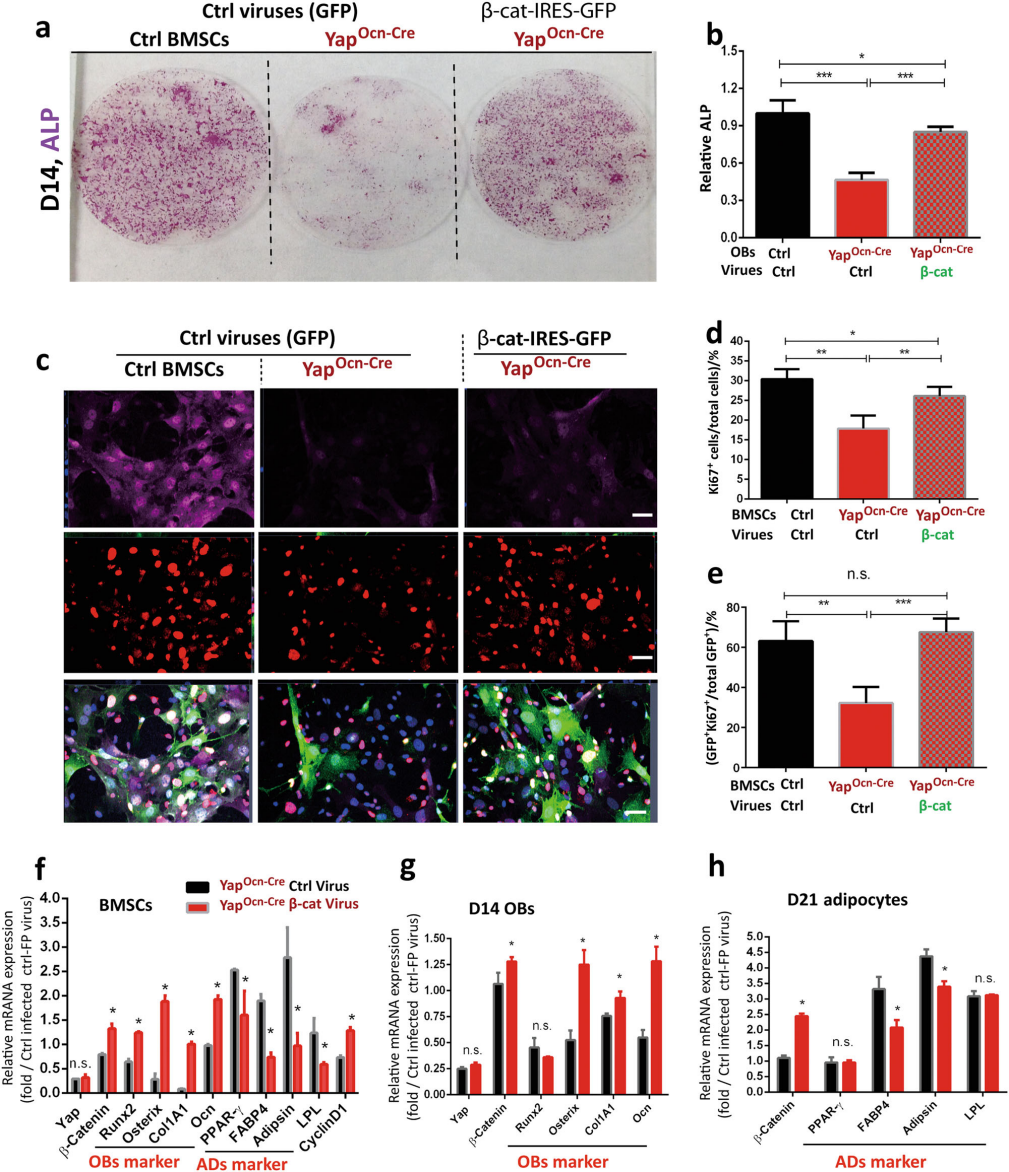
Rescue of OB genesis deficit by expression of β-catenin in Yap^Ocn-Cre^-BMSCs. Control and Yap^Ocn-Cre^ BMSCs (3-month mice) were infected with retroviruses encoding ctrl (GFP) and β-catenin-IRES-GFP. BMSCs were then subjected to OB (**a**, **b**) or immunostaining analysis for cell proliferation (**c**–**e**). At the indicated date of cultures, cells were stained for ALP enzymatic activity (**a**, **b**) or immunostained with anti-Ki67 antibody (**c**–**e**), respectively. Representative images were shown in **a**, **c**. Quantification data are presented in **b**, **d**, **e**. (Mean ± SD, *n* = 500 cells from 3 different cultures). *P < 0.05, **P<0.01, ***P<0.000 1. Scale bars: 20 μm. In **f**–**h**, real-time PCR analyses of the indicated genes’ expression were carried out in Yap^Ocn-Cre^ BMSCs, OBs (D14), and ADs (D21) infected with GFP and β-catenin retroviruses (mean ± SD, *n* = 3 different assays). *P < 0.05.

β-Catenin signaling is known to be critical for OB proliferation and differentiation.^15,21^ We thus speculate that YAP regulation of β-catenin may underlie YAP promotion of OB-proliferation and differentiation. Retroviruses encoding control green fluorescent protein (GFP) and β-catenin (β-catenin-IRES-GFP) were generated and used to infect control and Yap-deficient BMSCs (Supplemental Fig. 12A-C). Infected BMSCs were subjected to OB or adipocyte differentiation assays. At D14 cultures, Yap^Ocn-Cre^ BMSCs infected with control viruses showed lower ALP^+^ cells than that of WT controls, indicating a reduced OB differentiation (Fig. 6a, b). However, infection of the Yap-CKO BMSCs with the β-catenin viruses nearly completely restored OB differentiation, which was comparable to that of WT controls (Fig. 6a, b). Additionally, β-catenin overexpression in Yap-CKO BMSCs prevented adipocyte over-formation (Supplemental Fig. 12D, E) and restored their proliferation, as Ki67-marked proliferative BMSCs were much higher in Yap-CKO cultures infected with the β-catenin retroviruses than those of controls (Fig. 6c–e). Furthermore, β-catenin overexpression in Yap-CKO BMSCs/OB/ADs increased OB-associated gene expression (e.g., Runx2, osterix, and Col1A1, and osteocalcin) and cyclin D1 but decreased adipocyte-associated gene expression (e.g., PPARγ, FABP4, and adipsin) (Fig. 6f–h). Together, these results suggest that expression of β-catenin in Yap-deficient BMSCs is sufficient to restore OB genesis and to prevent adipocyte formation in culture, providing an evidence for YAP stabilization of β-catenin to underlie YAP regulation of OB genesis.

## Discussion

YAP, a key transcriptional factor downstream of the Hippo pathway, plays a crucial role in size control of multiple organs. Here we provide several lines of evidence for YAP’s function in maintaining bone mass. First, YAP is largely expressed in the OB lineage. Second, KO YAP in Ocn-Cre^+^ cells results in decreased bone formation, increased bone marrow fat, and Tb loss. Third, YAP-deficient BMSCs show impaired OB genesis and elevated adipogenic formation. Fourth, β-catenin is reduced in YAP-deficient BM-OB-lineage cells as well as in MC3T3 cells, and expression of β-catenin in Yap-deficient BMSCs diminished the OB genesis deficit. These results support a working model that YAP in OB-lineage cells promotes OB genesis and bone formation likely by interacting with and increasing β-catenin-mediated osteogenesis.

YAP regulation of adult Tb mass is evidence based on µCT and histological analyses of long bone structures of Yap-CKO mice, Yap^Ocn-Cre^. This function appears to be largely due to YAP promotion of OB-mediated bone formation, because bone formation is impaired, but bone resorption is unaffected, in Yap^Ocn-Cre^ mice (Figs. 2 and 3 and Supplemental Fig. 2). The reduced bone formation is in line with a recent report by Kegelman et al.,^22^ in which a reduced Tb mass and decreased bone formation are also detected in YAP and TAZ double CKO mice (by Osterix-Cre).^22^ However, in contrast from our result of normal bone resorption, they detect an increase of osteoclastic activity in the YAP and TAZ double CKO mice.^22^ Such a difference might be due to TAZ’s function and/or different Cre lines used. It is of interest to note that, although both Ocn-Cre and Osterix-Cre are expressed predominantly in OB-lineage cells, they also express in some non-OB-lineage cells in the bone marrow.^14,23^ However, for the non-OB-lineage cells in the bone marrow, the Osterix-Cre is detected in adipocytes and perivascular cells,^23^ where Ocn-Cre was not expressed (Supplemental Fig. 1).

Notice that the bone formation rate was not only reduced in Tb regions but also in endocortical bones in Yap^Ocn-Cre^ mice (Fig. 2). However, the cortical bone volume remains normal, inconsistent with the reduced endocortical bone formation. Such an inconsistency might be due to a compensatory increase of bone formation in the periosteum cortical region, thus resulting in a balanced cortical bone volume, with increased Ec. and Ps. perimeters in the cortical bones of Yap-CKO mice (Fig. 2o, r–u). In line with this speculation, more Ocn-Cre-driven Td-positive OBs/osteocytes (Supplemental Fig. 6E, F), BrdU^+^ cells (likely to be OBs) (Fig. 4m, n), and elevated bone formation (Fig. 2o, t, u) were detected in the periosteum region of the Yap-CKO cortical bones, compared with that of controls. Interestingly, inhibition of Wnt/β-catenin signaling in osteocytes also results in an elevated periosteum osteoblastic proliferation.^24^

YAP promotion of osteogenesis is in agreement with the reports of TAZ’s function in this event.^22,25,26^ In fact, TAZ may play a compensatory effect in Yap-CKO mice, thus we detected ~50%–80% reduction in Ocn-Cre^+^ cells or OB differentiation when YAP is depleted. However, controversial reports exist regarding YAP’s function in this event.^7,11,27^ Seo et al. have reported that YAP is a direct target of SOX2, but osteogenesis is suppressed by high SOX2 or YAP1 and increased by depletion of either SOX2 or YAP1 in MSCs or C3H10T1/2 cells in culture.^27^ They also found that both overexpression or underexpression of YAP in these cells inhibits adipogenesis in vitro.^27^ Thus they propose that SOX2-YAP signaling induces Wnt antagonist Dkk1 to diminish osteogenic signaling in favor of adipogenesis.^27^ Obviously, our results disagree with this view, as DKK1 is unchanged in Yap-CKO BMSCs and OBs culture compared with their controls (Supplemental Fig. 7G-I). However, our results support the notion for YAP as a target of SOX2, because OB-specific SOX2-CKO results in similar bone deficits as that of Yap^Ocn-Cre^ mice: lower bone density, cell senescence in osteoprogenitors, and reduced bone formation.^28^ The exact reason(s) for the controversial results regarding YAP regulation of osteogenesis/adipogenesis remains unclear, which might have resulted from different MSCs from different age groups of mice examined.

Notice that, in addition to the OB-lineage, Ocn-Cre-driven Td was detected in fractional non-OB-lineage cells, including chondrocytes in the GP (~30%), CXCL12^+^ CAR-like cells (~30%), and NG2^+^ pericytes (~15%) in the bone marrow (Fig. 1f, i–k and data not shown), in line with the report by Zhang and Link,^14^ and raising a concern about the specificity of the Ocn-Cre driver. Although Ocn-Cre is expressed in fractions of non-OB-lineage cells, the following observations led us to believe that YAP in the OB lineage plays a critical role in promoting OB genesis. First, Ocn-Cre is largely expressed in the OB lineage, with nearly 100% of OB-lineage cells (including OBs in the Tb, lining cells, and osteocytes in the cortical bone) marked by Ocn-Cre-driven Td (Fig. 1i–k, and Supplemental Fig. 2). In cultured BMSCs, although Ocn-Cre-driven Td over total BMSCs was low ( < 30%)(Figs. 1 and 4), upon differentiation with proper factors, these cells committed to ALP^+^ OBs, but not to anti-perilipin-marked adipocytes (Supplemental Fig. 1). Second, BMSCs derived from Yap^Ocn-Cre^ mice exhibited impaired OB genesis (Fig. 3), eliminating a possible role of YAP in NG2^+^ cells in this event, as NG2^+^YAP^+^ cells were undetectable in this type of cultures (Supplemental Fig. 1G and data not shown). Third, Ocn-Cre^+^ cells in BMSCs were increased upon in vitro OB differentiation (Fig. 1e and Supplemental Fig. 1D, E) and decreased when Yap was knocked out (Fig. 4a–e). Fourth, the decrease of Ocn-Cre^+^ cells in BMSC cultures correlated well with the deficits of both in vitro OB genesis and in vivo bone formation. Considering all of these observations, we propose that the defects in both in vitro OB genesis and in vivo assays are largely due to YAP depletion in Ocn-Cre^+^ OB-lineage cells.

Whereas our results suggest a critical function of YAP in Ocn-Cre^+^ OB-lineage cells for OB genesis, the functions of YAP in Ocn-Cre^+^ non-OB-linage cells remain unclear. It remains a possibility for YAP in these cells to negatively regulate adipocyte formation from BMSCs. It is also possible for YAP in these cells (e.g., chondrocytes) to regulate chondrocyte’s function, given that Yap1 deletion in Col2a1-Cre^+^ cells impairs early chondrocyte proliferation but increases subsequent maturation.^29^ However, our preliminary examinations of GP structure (by H&E staining) and function (by Safranin O staining) showed no obvious alteration in Yap^Ocn-Cre^ mice (data not shown). Thus these results highlight the need for a further investigation of YAP in Ocn-Cre^+^ non-OB-lineage cells’ effect on chondrogenesis, osteogenesis, and adipogenesis.

How does YAP in the OB lineage promote osteogenesis? We hypothesize that YAP stabilizes β-catenin and thus increases nuclear β-catenin-mediated osteogenesis. This is supported by our results that YAP co-expressed with β-catenin in OB-lineage cells (Fig. 1); YAP-deficiency in OBs reduces cytoplasmic and nuclear β-catenin levels in culture and in vivo (Figs. 4c, f and 5 and Supplemental Fig. 7-11); expression of YAP in Yap-KO MC3T3 cells increased β-catenin (Fig. 5k, l); and expression of β-catenin in Yap-deficient BMSCs could diminish OB genesis deficit (Fig. 6). Whereas our results favor the model for YAP as a positive regulator of Wnt/β-catenin signaling and osteogenesis, YAP/TAZ has been reported to play both positive and negative roles in Wnt signaling.^8,30–32^ For example, YAP is required for stabilization of Smad1/5/8 but not β-catenin in astrocytes in the brain.^8^ Park et al. report that overexpression of YAP-5SA mutant in MCF10A cells (a breast cancer cell line) decreases β-catenin levels and inhibits Wnt signaling^32^ thus conclude a negative role of YAP/TAZ on Wnt/β-catenin signaling. In contrast to this report, we found that expression of WT YAP in MC3T3 cells increased β-catenin (in both cytoplasmic and nuclear pools) (Fig. 5k, l). These controversial results may be due to different cell lines used. YAP may regulate β-catenin not only at the cytoplasmic level but also in the nucleus, in a cell-type-dependent manner. These observations also highlight a need for further investigation of YAP regulation of Wnt/β-catenin signaling. In addition to YAP-β-catenin signaling, other mechanisms may underlie YAP regulation of osteogenesis. It has been reported that YAP and TAZ associate with SMADs to promote transcription of transforming growth factor-β and BMP target genes^4,10,33^ and functions,^8,34^ and treatment of MSCs with BMP2 leads to increased YAP/TAZ expression^8,34^ and enhanced interaction with RUNX2 to promote OB differentiation.^25^ As phospho-Smad1/5/8 is unchanged in YAP-deficient OB-lineage cells, we believe that YAP-β-catenin is a major mechanism underlying YAP regulation of osteogenesis.

How does YAP in Ocn-Cre^+^ cells suppress adipogenesis? In light of reports that TAZ plays a negative role in adipogenesis likely by interacting and suppressing PPARγ (a key transcription factor orchestrating adipogenesis),^25^ and treatment with a ligand (the small molecule KR62980) for PPARγ that antagonizes adipocyte differentiation promotes TAZ nuclear localization and enhances interaction between TAZ and PPARγ,^35^ we speculate that YAP may act as TAZ, interacting with PPARγ and suppressing its activity in adipogenesis as PPARγ was increased in Yap-depleted BMSCs. Whereas this speculation remains to be further tested in future, other possibility exists. For examples, YAP in Ocn-Cre^+^ OB-lineage cells may regulate adipocyte differentiation from MSCs in a cell-non-autonomous manner. A negative factor for adipogenesis may be released from OB progenitors, which is regulated by YAP. Alternatively, YAP in Ocn-Cre^+^ non-OB-lineage cells (e.g., CXCL12^+^ CAR cells) may play a role in suppressing adipogenesis. Thus multiple mechanisms may account for YAP regulation of adipogenesis, which requires further investigation.

## Materials and methods

### Animals

Yap^Ocn-Cre^ CKO mice were generated by crossing floxed Yap allele (Yap^f/f^) with osteocalcin (Ocn)-Cre transgenic mice (provided by Dr. T. Clemens, Johns Hopkins Medical School). Ocn-Cre; Ai9 and Yap^Ocn-Cre^; Ai9 mice were generated by crossing Ai9 mice (from the Jackson Laboratory, donated by Dr. Hongkui Zeng, Allen Institute for Brain Science) with Ocn-Cre and Yap^Ocn-Cre^ mice, respectively. Ai9 mice have a loxP-flanked STOP cassette preventing translation of a CAG promoter-driven red fluorescent protein variant (tdTomato). Yap^Ocn-Cre^; Axin2-LacZ mice were generated by crossing Axin2-LacZ (from the Jackson laboratory, donated by Dr. Walter Birchmeier, Max-Delbrueck-Center) with Yap^Ocn-Cre^ mice. Axin2-LacZ mice have LacZ expression under the control of Axin2 promoter, which depends on Wnt/β-catenin signaling. Both male and female mice were characterized, and Yap-CKO mice (both male and female) showed similar phenotypes. All mice, maintained in C57BL/6 background, were housed in a room with a 12 h light/dark cycle with ad libitum access to water and rodent chow diet (Diet 7097, Harlan Teklad). The use of experimental animals has been approved by the IACUC (Institutional Animal Care and Use Committee) at Augusta University and Case Western Reserve University in accordance with NIH guidelines.

### Statistical analysis

All data were expressed as mean ± SD. For in vivo studies (e.g., microCT, histomorphometry examinations, and bone dynamic histomorphometric analysis), 5–6 mice per genotype per assay per sex were used. For cell culture experiments, each experiment used 1–2 mice/genotype and was repeated for 3 times and thus *n* = 3–5 mice/genotype/assay. For quantification analysis of fluorescence intensity in immunostained primary cultured cells, 10–50 cells were examined. For Td^+^ cells in vivo and in vitro, ~200 cells were examined, which were from 3 to 5 different mice/genotype. Both Student's *t*-test and one-way anaysis of variance (GraphPad Prism 6) were used, and the significance was set at *P* < 0.05.

## Electronic supplementary material

Supplemental information

## References

[1] Zhao B, Li L, Lei Q, Guan KL (2010). The Hippo-YAP pathway in organ size control and tumorigenesis: an updated version. Genes Dev..

[2] Piccolo S, Dupont S, Cordenonsi M (2014). The biology of YAP/TAZ: hippo signaling and beyond. Physiol. Rev..

[3] Camargo FD (2007). YAP1 increases organ size and expands undifferentiated progenitor cells. Curr. Biol..

[4] Varelas X (2010). The Hippo pathway regulates Wnt/beta-catenin signaling. Dev. Cell.

[5] Pan D (2010). The hippo signaling pathway in development and cancer. Dev. Cell.

[6] Mo JS, Park HW, Guan KL (2014). The Hippo signaling pathway in stem cell biology and cancer. EMBO Rep..

[7] Dupont S (2011). Role of YAP/TAZ in mechanotransduction. Nature.

[8] Huang Z (2016). YAP stabilizes SMAD1 and promotes BMP2-induced neocortical astrocytic differentiation. Development.

[9] Azzolin L (2014). YAP/TAZ incorporation in the beta-catenin destruction complex orchestrates the Wnt response. Cell.

[10] Alarcon C (2009). Nuclear CDKs drive Smad transcriptional activation and turnover in BMP and TGF-beta pathways. Cell.

[11] Zaidi SK (2004). Tyrosine phosphorylation controls Runx2-mediated subnuclear targeting of YAP to repress transcription. EMBO J..

[12] Huang Z (2016). YAP is a critical inducer of SOCS3, preventing reactive astrogliosis. Cereb. Cortex.

[13] Zhang M (2002). Osteoblast-specific knockout of the insulin-like growth factor (IGF) receptor gene reveals an essential role of IGF signaling in bone matrix mineralization. J. Biol. Chem..

[14] Zhang J, Link DC (2016). Targeting of mesenchymal stromal cells by cre-recombinase transgenes commonly used to target osteoblast lineage cells. J. Bone Miner. Res..

[15] Day TF, Guo X, Garrett-Beal L, Yang Y (2005). Wnt/beta-catenin signaling in mesenchymal progenitors controls osteoblast and chondrocyte differentiation during vertebrate skeletogenesis. Dev. Cell.

[16] Chen G, Deng C, Li YP (2012). TGF-beta and BMP signaling in osteoblast differentiation and bone formation. Int. J. Biol. Sci..

[17] Dong YF (2006). Wnt induction of chondrocyte hypertrophy through the Runx2 transcription factor. J. Cell. Physiol..

[18] Spencer GJ (2006). Wnt signalling in osteoblasts regulates expression of the receptor activator of NFkappaB ligand and inhibits osteoclastogenesis in vitro. J. Cell Sci..

[19] Shtutman M (1999). The cyclin D1 gene is a target of the beta-catenin/LEF-1 pathway. Proc. Natl. Acad. Sci. USA.

[20] Yu HM (2005). The role of Axin2 in calvarial morphogenesis and craniosynostosis. Development.

[21] Baron R, Kneissel M (2013). WNT signaling in bone homeostasis and disease: from human mutations to treatments. Nat. Med..

[22] Kegelman, C. D. et al. Skeletal cell YAP and TAZ combinatorially promote bone development. *FASEB* J.fj201700872R (2018). 10.1096/fj.201700872R PMC5901392 29401582

[23] Chen J (2014). Osx-Cre targets multiple cell types besides osteoblast lineage in postnatal mice. PLoS ONE.

[24] Moon YJ (2016). Maturation of cortical bone suppresses periosteal osteoprogenitor proliferation in a paracrine manner. J. Mol. Histol..

[25] Hong JH (2005). TAZ, a transcriptional modulator of mesenchymal stem cell differentiation. Science.

[26] Yang JY (2013). Osteoblast-targeted overexpression of TAZ increases bone mass in vivo. PLoS ONE.

[27] Seo E (2013). SOX2 regulates YAP1 to maintain stemness and determine cell fate in the osteo-adipo lineage. Cell Rep..

[28] Basu-Roy U (2010). The transcription factor Sox2 is required for osteoblast self-renewal. Cell Death Differ..

[29] Deng Y (2016). Yap1 regulates multiple steps of chondrocyte differentiation during skeletal development and bone repair. Cell Rep..

[30] Zhao K (2017). Muscle Yap is a regulator of neuromuscular junction formation and regeneration. J. Neurosci..

[31] Heallen T (2011). Hippo pathway inhibits Wnt signaling to restrain cardiomyocyte proliferation and heart size. Science.

[32] Park HW (2015). Alternative Wnt signaling activates YAP/TAZ. Cell.

[33] Varelas X (2010). The Crumbs complex couples cell density sensing to Hippo-dependent control of the TGF-beta-SMAD pathway. Dev. Cell.

[34] Huang Z (2016). Neogenin promotes BMP2 activation of YAP and Smad1 and enhances astrocytic differentiation in developing mouse neocortex. J. Neurosci..

[35] Jung H (2009). Augmentation of PPARgamma-TAZ interaction contributes to the anti-adipogenic activity of KR62980. Biochem. Pharmacol..

