# Supplementary material for: YAP promotes osteogenesis and suppresses adipogenic differentiation by regulating β-catenin signaling
Source: Bone Res. 2018 Jun 1;6:18. doi: 10.1038/s41413-018-0018-7 (PMC5984632; doi:10.1038/s41413-018-0018-7)
Supplement: Supplementary file 1 — Supplemental information [file 41413_2018_18_MOESM1_ESM.docx]

**YAP Promotes Osteogenesis and Suppresses Adipogenic Differentiation by Regulating β-Catenin Signaling**

**SUPPLEMENTAL FIGURES**

**Supplemental Fig. 1.**


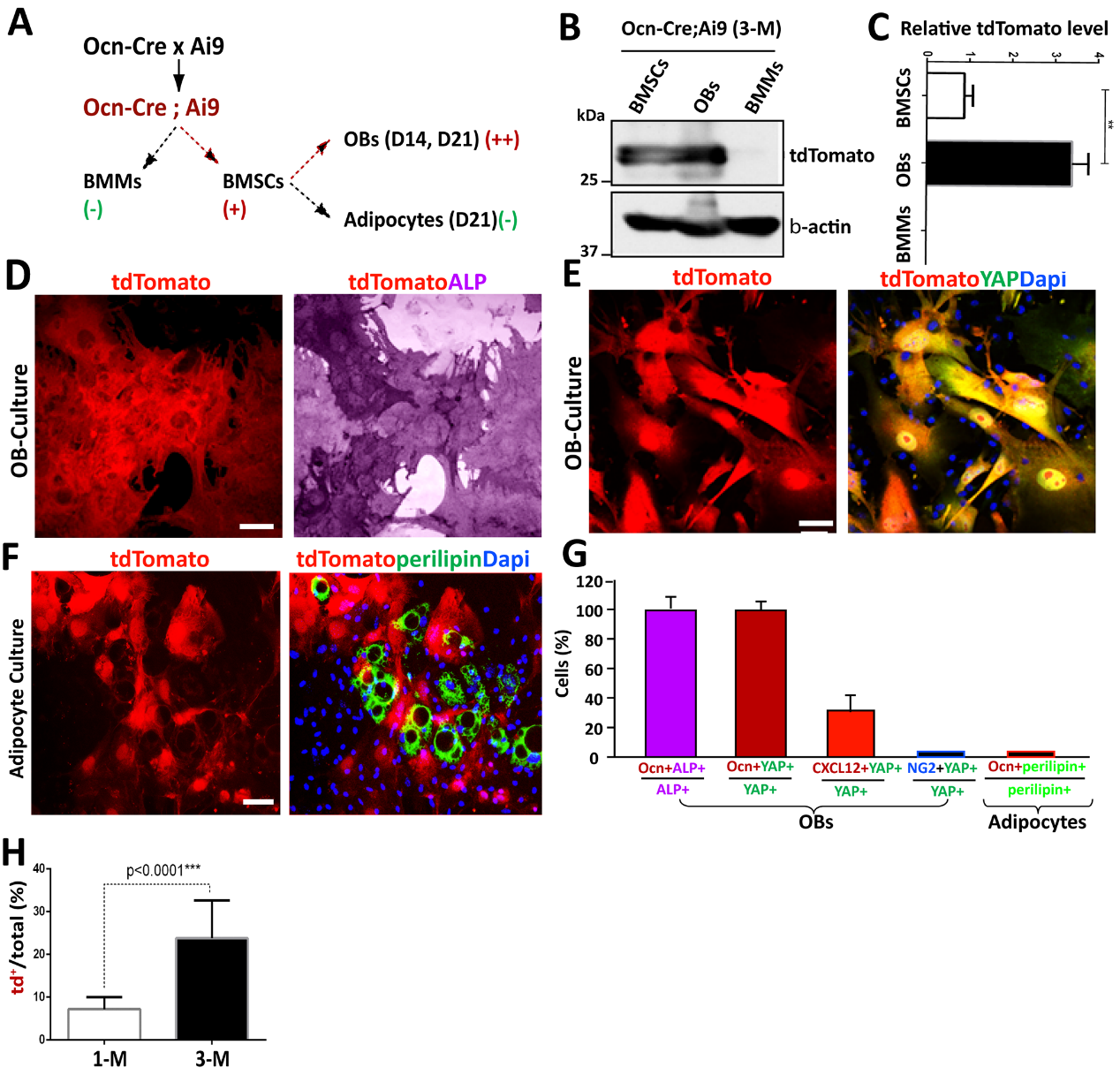


Supplemental Fig. 1. Generation of Ocn-Cre; Ai9 mice and co-expression of YAP with tdTomato in OB-, but not adipocytes, nor BMMs. (A) Illustration of the generation of Ocn-Cre; Ai9 mice and cultured bone cells (BMSCs, OBs, BMMs and adipocytes) from Ocn-Cre; Ai9 mice. (B-C) Western blot analysis of tdTomato expression in BMSCs, OBs, and BMMs. (D) Co-staining of ALP and tdTomato in OBs (D14 culture) differentiated from BMSCs. (E) Immunostaining analysis of YAP in tdTomato^+^ OBs (D14 culture). (F) Immunostaining analysis of Perilipin in adipocytes (D21 culture) differentiated from BMSCs. (G) Quantification analyses of data from (D-F). (Mean ± SD, n =50 from 3-different cultures). *, P < 0.05. In (B-G), BMSCs or BMMs were from 3-M old Ocn-Cre; Ai9 mice, and OBs/adipocytes were differentiated from BMSCs under proper soluble factors in culture (see Supplemental Methods), Scale bar, 20 µm. (H) Quantification analysis of percentage of Td^+^ cells in 1-M and 3-M BMSCs culture. The values of mean ± SD (n = 20) from 3-independent assays were presented. *, P < 0.05.

**Supplemental Fig. 2.**


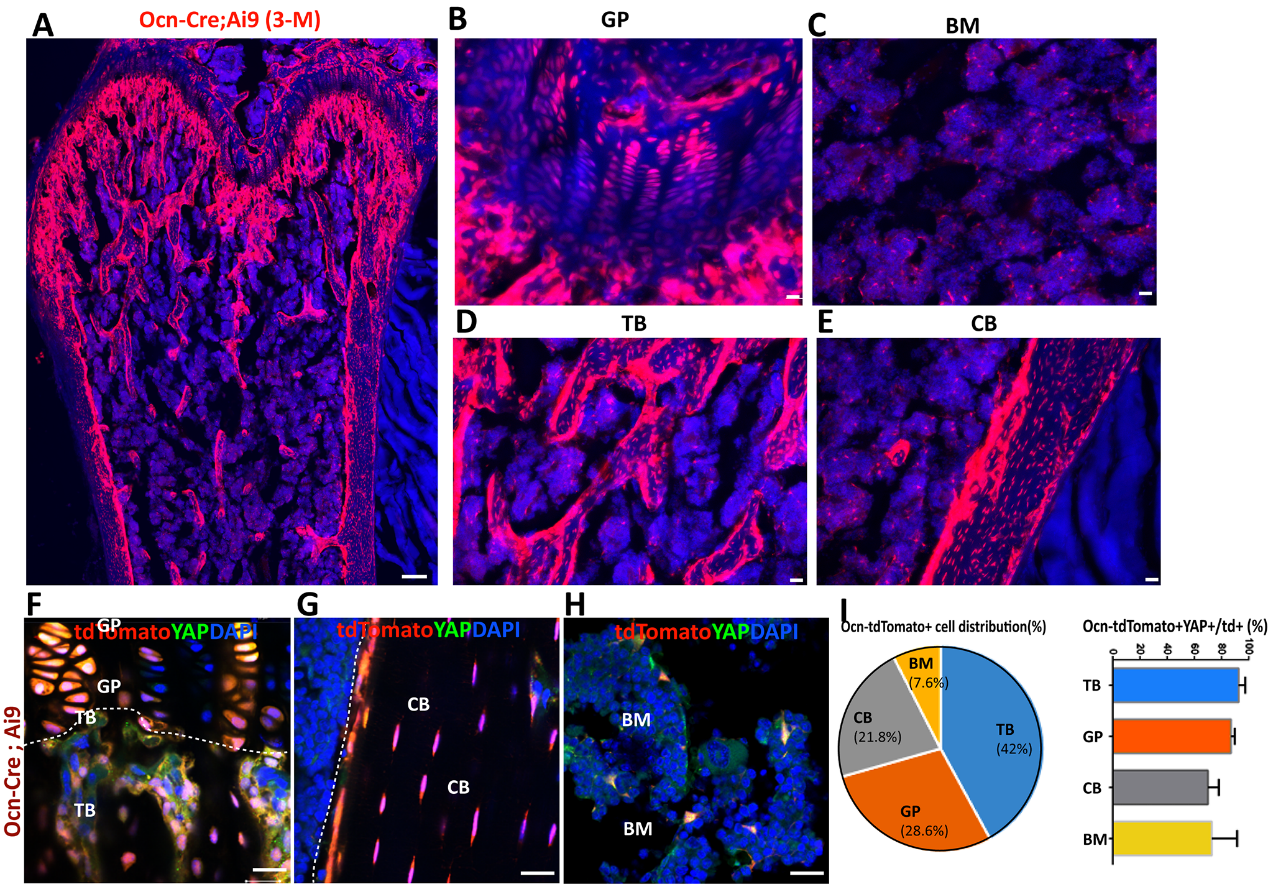


Supplemental Fig. 2. TdTomato distribution and immuno-fluorescence staining analysis of YAP in femur bone sections from Ocn-Cre;Ai9 mice (3-M old). (A-E) TdTomato fluorescence imaging in femur bone section from Ocn-Cre;Ai9 mice (3-M old). Higher power magnifications of GP, BM, TB and CB area of (A) are shown in (B-E). Scar bar in (A) is 100µm, and Scale bars in (B-E) are 20µm. (F-H) Immune-fluorescence staining analyses of YAP with tdTomato in femur bone sections from Ocn-Cre; Ai9 mice (3-M old). Representative co-focal images were shown in (F-H); Scale bar 10µm. (I) Quantification analyses. (Td^+^ fluorescence intensity was measured in TB, GP, CB and BM area of femurs (shown in A) by use of NIH Image J software. The whole bone defined as 100%. Therefore, % means percentage of each area. Based on distribution of Td^+^ cells, they likely to be OB-lineage cells including osteoblast, osteocytes, lining cells, osteoblasts in the periosteum.) (Mean ± SD, n =5). TB, trabecular bone; CB, cortical bone; BM, bone marrow; and GP, growth plate.

**Supplemental Fig. 3.**


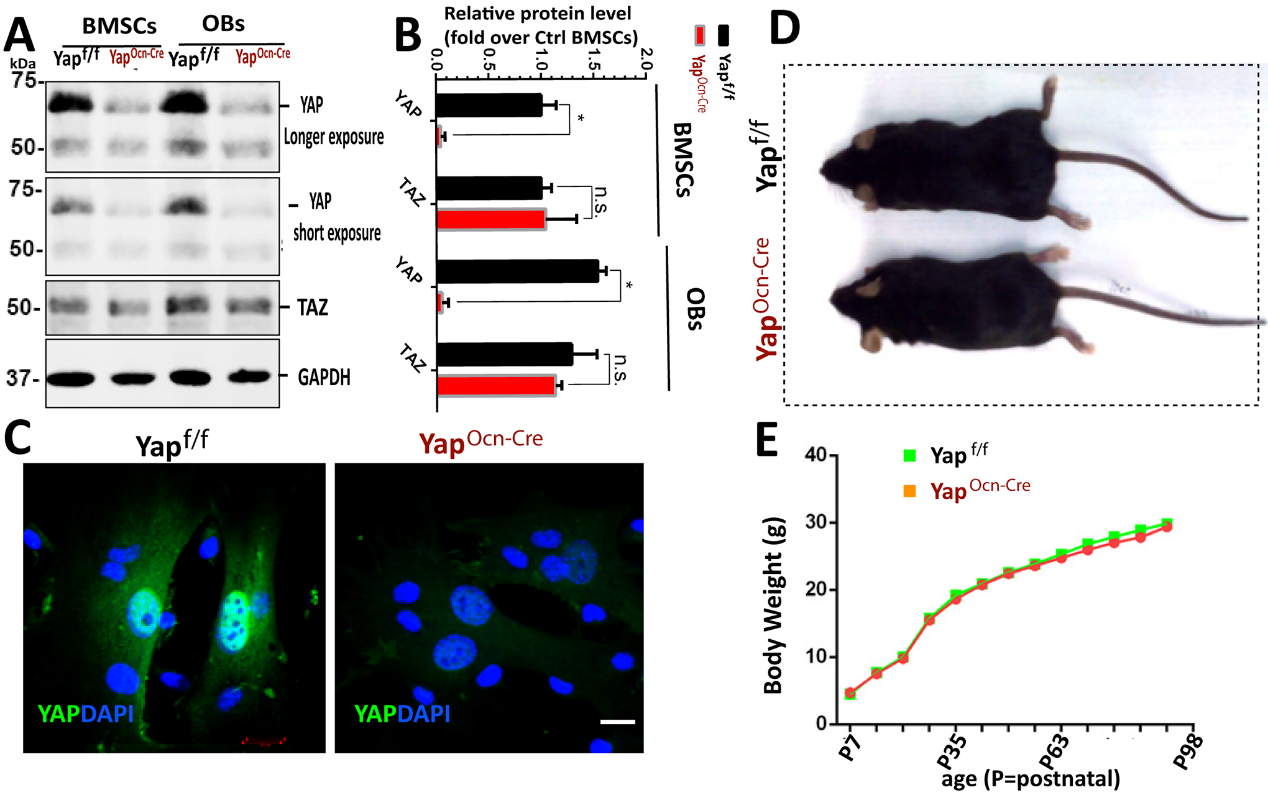


Supplemental Fig. 3. Generation of Yap^Ocn-Cre^ mice. (A-B) Western blot analysis of YAP and TAZ expression in lysates of OBs and BMMs derived from control (Yap^f/f^) and Yap^Ocn-Cre^ mice. Antibodies (YAP, WH0010413M1, Sigma; TAZ, T4077, Sigma) were used. The data were quantified by use of NIH Image J software and presented in (B) (mean ± SD, n =3-different cultures). *, P < 0.05. (C) Immunostaining analysis of YAP in BMSCs from control and Yap^Ocn-Cre^ mice. Scale bar 20µm. (D) Images of Yap^f/f^ and Yap^Ocn-Cre^ mice. (E) Normal body weights in Yap^Ocn-Cre^ mice, compared to that of control mice.

**Supplemental Fig. 4.**

**
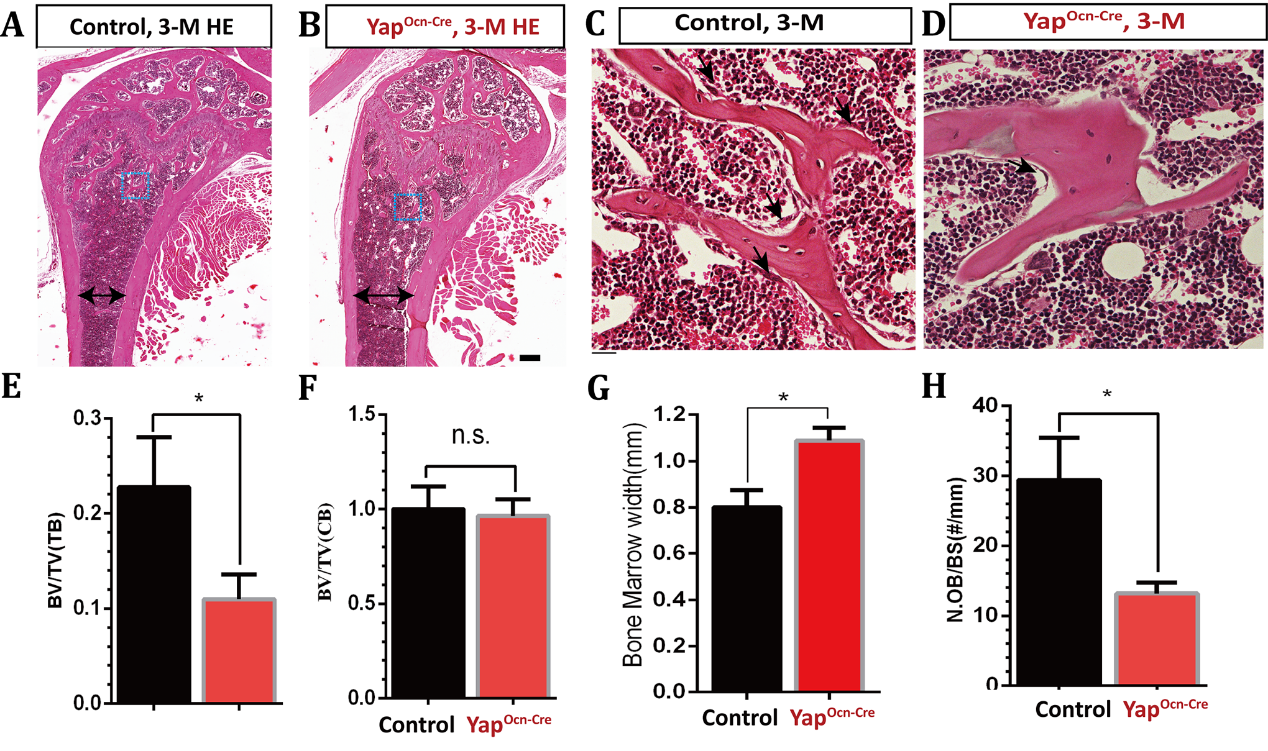
**

**Supplemental Fig. 4. Trabecular bone-loss in female Yap^Ocn-Cre^ mice (3-M old)**

H & E staining analysis of femurs from 3-M old female ctrl and Yap^Ocn-Cre^ mice showed decreases in bone mass and OB numbers in TB, and an increase in bone marrow width, but normal bone volume in CB in Yap^Ocn-Cre^ mice. (**A-D)** Representative images, Scar bar 300µm, C, D amplified images of A, B (marked blue squares), respectively. (**E-F)** Quantification analyses of BV/TV in TB and CB, respectively. **(G)** Quantification analysis of bone marrow width. **(H)** Quantification analysis of OB numbers per trabecular bone surface. In E-H, data are shown as mean ± SD, n = 5 female mice. *, P < 0.05.

**Supplemental Fig. 5.**


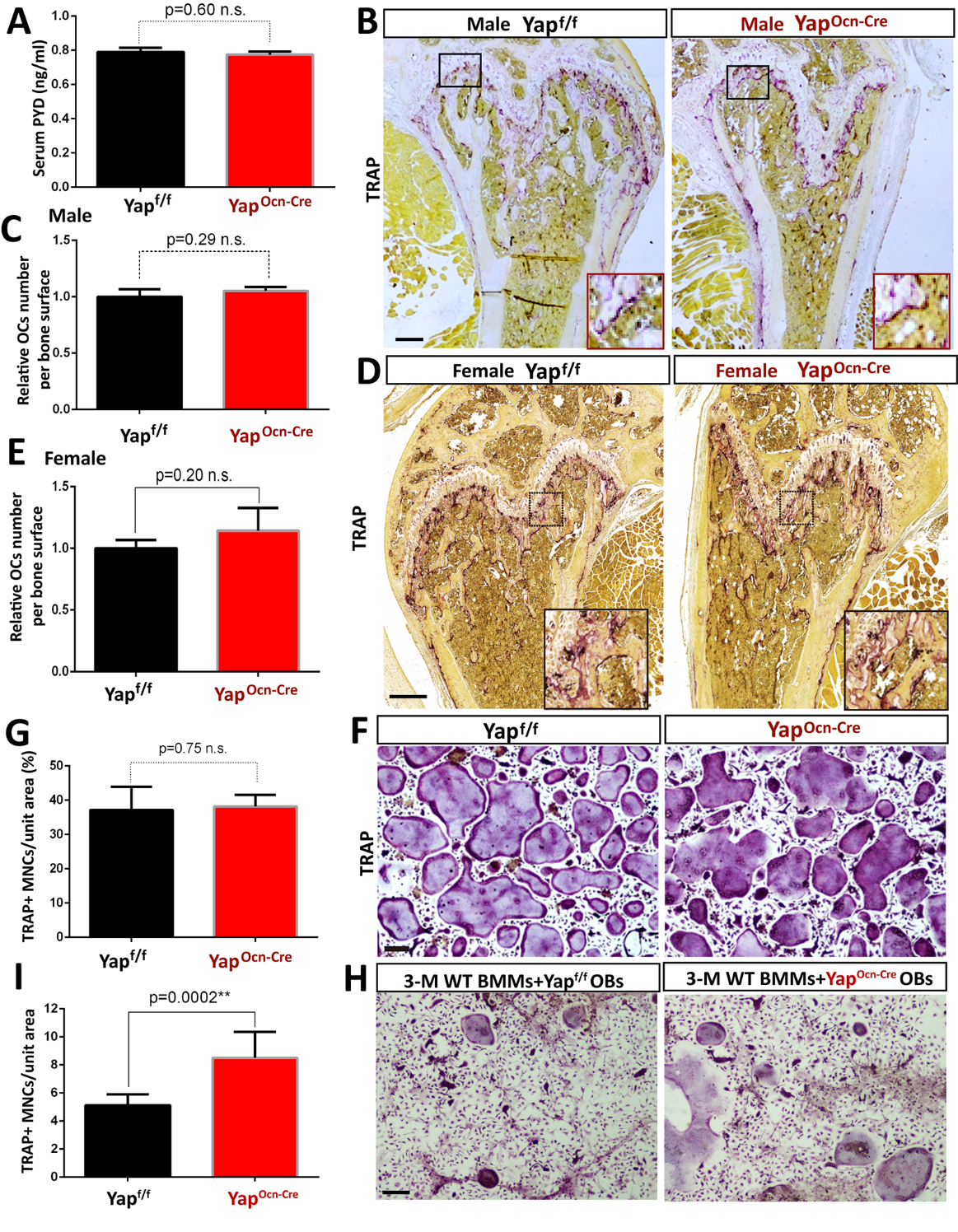


**Supplemental Fig. 5. Normal bone resorption and TRAP staining analysis in Yap^Ocn-Cre^** **mice.**

**(A)** Serum levels of PYD in control and Yap^Ocn-Cre^ mice (3-months old, male). **(B-E)** TRAP staining analysis of femur sections from 3-months old control and Yap^Ocn-Cre^ male and female mice. Representative images were shown in (B, D), Scale bar 200µm. Images marked with black squares were amplified and showed in the bottom panels. The quantitative analysis of OCs number per unit bone surface (BS) in male and female mice was carried out in trabecular bones of femurs and showed in (C, E). The values of mean ± SD from 3 different animals were presented. **(F-G)** In vitro OC genesis of BMMs derived from control and Yap^Ocn-Cre^ mice (3-months old). OCs were generated from purified BMMs (5 x 10^4^) cultured in the presence of RANKL (100 ng/ml) and M-CSF (10ng/ml) for 6 days. Representative images of TRAP staining were shown in (F), Scale bar 100µm. Quantitative analyses of the average TRAP positive multi-nuclei cell (MNC) density [count TRAP+ MNCs (>3 nuclei per cell) per unit area] were presented in (G). The values of mean ± SD from 3 separate cultures were shown. **(H-I)** TRAP staining analysis of multiple nuclei cells (MNCs or OCs) derived from WT BMMs co-culture with OBs from Control and Yap^Ocn-Cre^ mice. WT BMMs were seeded and mixed cultured with OBs with αMEM supplemented with 10% FBS and 10^−8^ M 1, 25 dihydroxyvitamin D. The co-cultures were grown for 10 days and subjected for TRAP staining. Representative images are shown in H, the quantitative analyses of TRAP^+^ MNCs over total cells are presented in I. The values of mean ± SD from 3-different experiments were presented.

**Supplemental Fig. 6.**

**
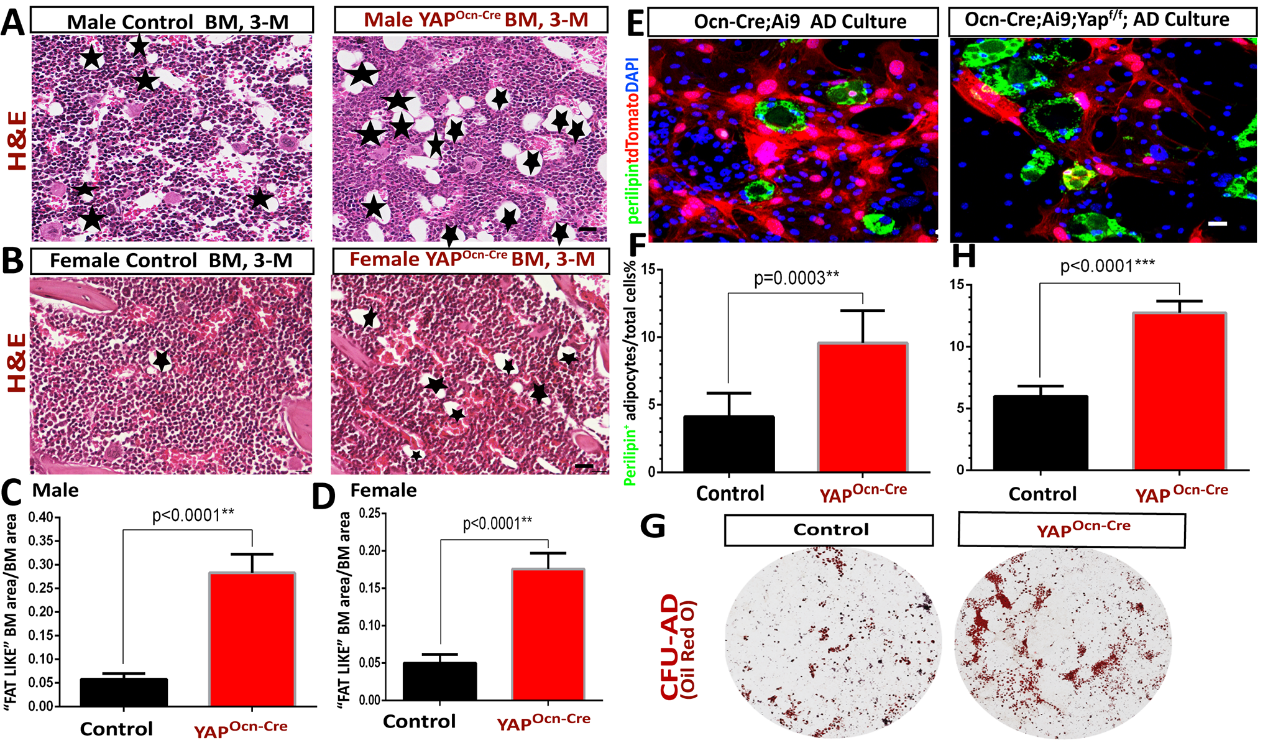
**

**Supplemental Fig. 6. Increase bone marrow fat in Yap^Ocn-Cre^ mice (3-M old) and increases adipocyte differentiation in Yap^Ocn-Cre^-BMSC/OB cultures.** **(A-D)** H & E staining analysis of femurs from 3-M old ctrl and Yap^Ocn-Cre^ mice (both male and female) showed an increase in “Fat-like” cells in the Yap^Ocn-Cre^ mice. A, B, representative images, Scar bar 20µm. In C and D, quantification analysis of “fat-like” vacuoles (area) over BM area in male and female mice separately. (mean ± SD, n = 5). *, P < 0.05. **(E-F)** Immunostaining analysis using anti-perilipin of adipocytes differentiated from BMSCs of Ocn;Ai9 and Ocn; Ai9; Yap^f/f^ mice. E, representative images, Scale bar 20µm; and F, quantification analysis (mean ± SD, n =500 cells). *, P < 0.05. **(G-H)** CFU-AD was increased in Yap^Ocn-Cre^-BMSC cultures. CFU-AD assays were described in the supplemental material. G, Oil Red O staining image and the quantification (mean ± SD, n =3 different cultures) was shown in (H).

**Supplemental Fig. 7.**


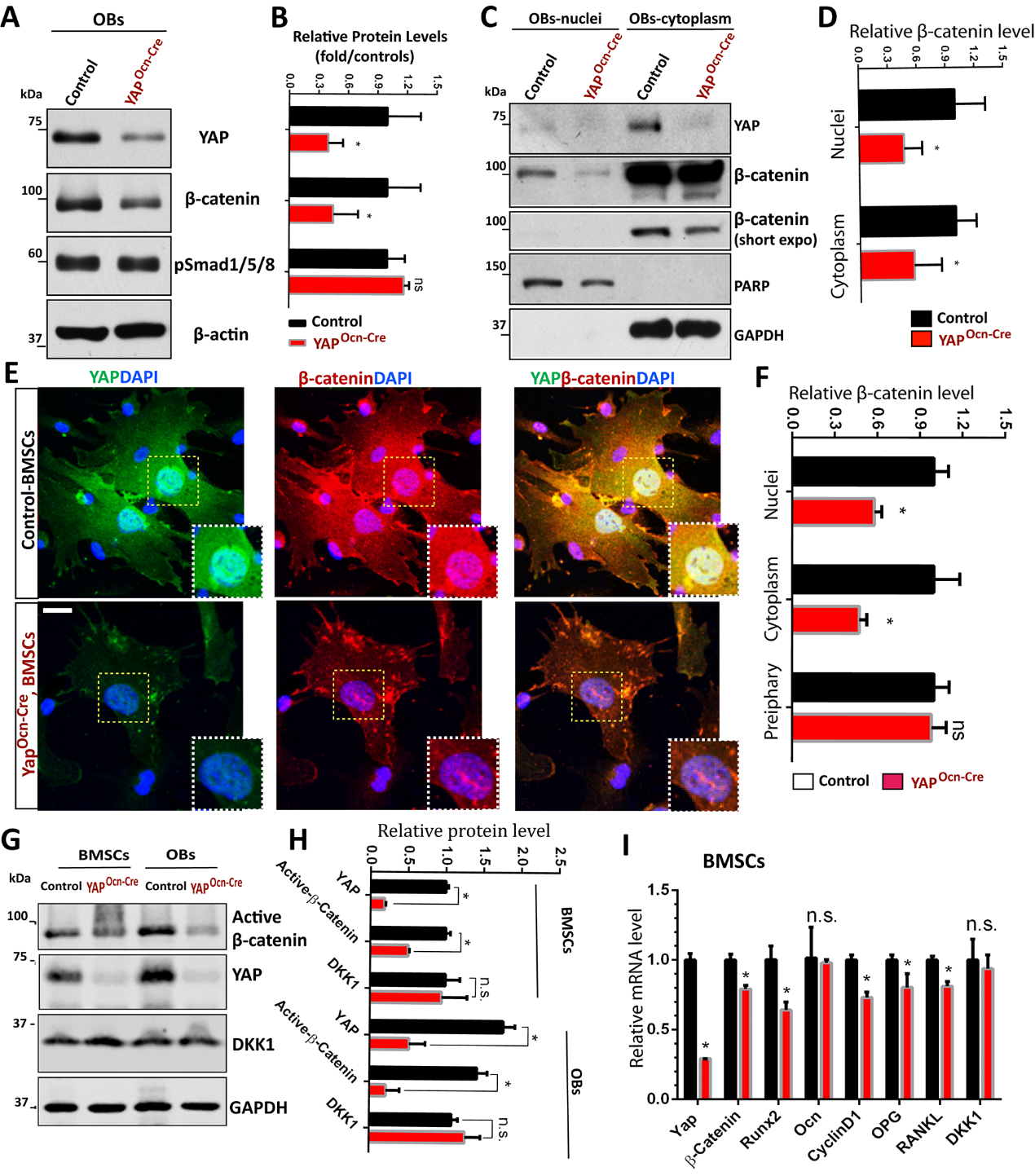


**Supplemental Fig. 7. Decreases in cytoplasmic and nuclear β-catenin levels in Yap^Ocn-Cre^** **OB-lineage cells. (A-D)** Western blot analysis of β-catenin levels. Total lysates (A-B) and lysates of nuclei and cytoplasmic fractions (C-D) of OBs (D14 culture from BMSCs) were subjected to the immunoblot analysis using indicated antibodies (YAP, WH0010413M1, Sigma; β-catenin, C2206, Sigma). Representative blots were shown in (A, C). The data were quantified by use of NIH Image J software and presented in (B, D) (mean ± SD, n =3-different cultures). *, P < 0.05.

**(E-F)** Co-immunostaining analysis of β-catenin and YAP. BMSCs from Ocn-Cre; Ai9 and Ocn-Cre; Ai9; Yap^f/f^ mice were fixed and subjected to co-immunostaining analysis using indicated antibodies (β-catenin C7207, Sigma; YAP #4912, CST). Representative images were shown in (E), scale bar, 50µm, and the quantification data were presented in (F) (mean ± SD, n =20 from 3-different cultures). *, P < 0.05. **(G-H)** Western blot analysis of active β-catenin and DKK1 levels in BMSCs and OBs. Total lysates of BMSCs and OBs ((D14 culture differentiated from BMSCs)) were subjected to the immunoblot analysis using indicated antibodies. Representative blots were shown in (G). The data were presented in (H) (mean ± SD, n =3-different cultures). *, P < 0.05. **(I)** RT-PCR analysis of Wnt target gene and the Wnt inhibitor DKK1 expression in primary culture BMSCs. The data were present as mean ± SD (n =3-different cultures). *, P < 0.05.

**Supplemental Fig. 8.**


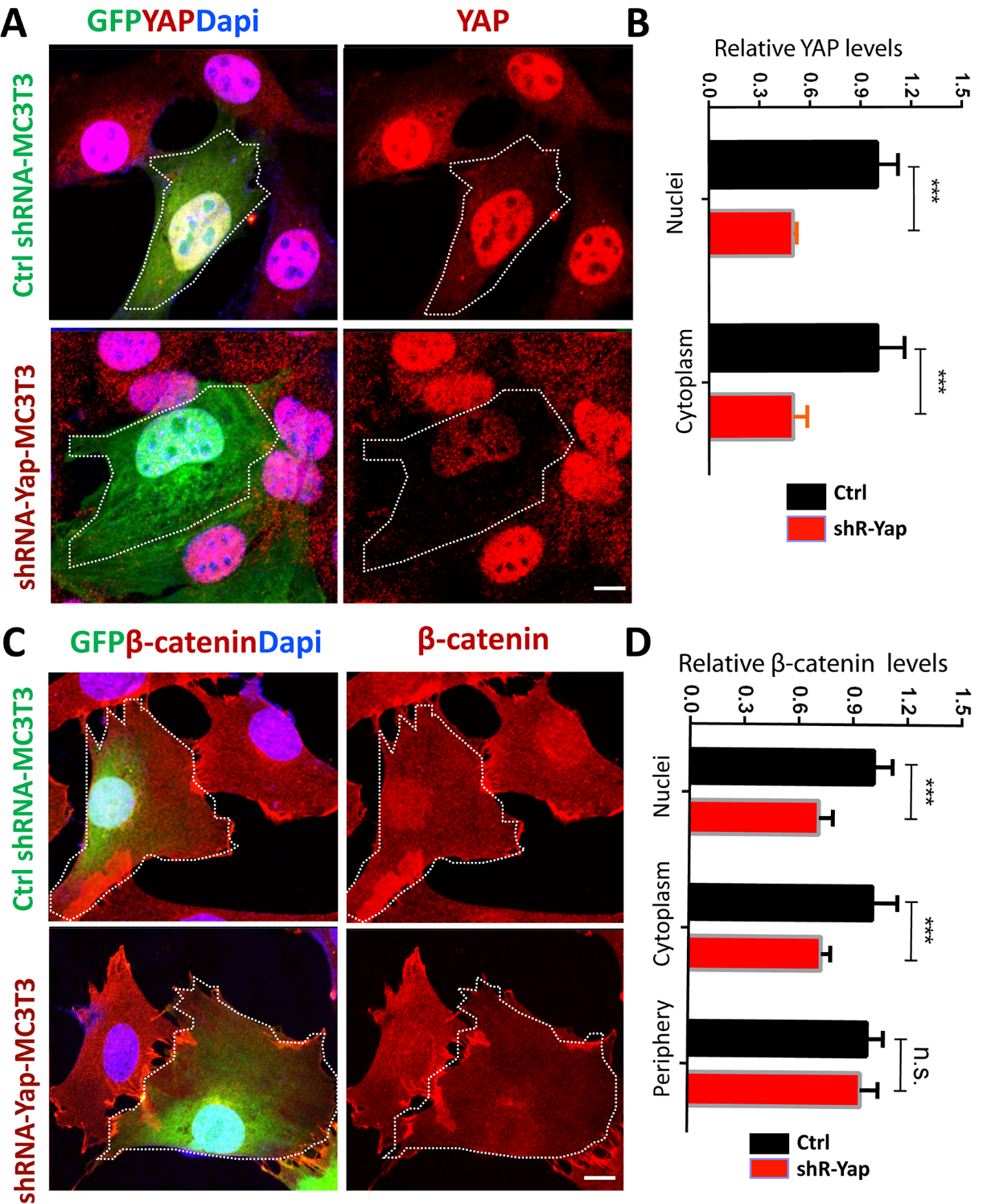


**Supplemental Fig. 8. Reduction of β-catenin in MC3T3 cells expressing shRNA-Yap.** MC3T3 cells were transiently transfected with control (GFP) and shRNA-Yap plasmids. 4-days after transfection, cells were fixed and subjected to immunostaining analyses using indicated antibodies (YAP, WH0010413M1, Sigma; β-catenin, C2206, Sigma). **(A-B)** Reduction of YAP in MC3T3 cells expressing shRNA-Yap. **(C-D)** Decreases in cytoplasmic and nuclear levels of β-catenin in MC3T3 cells expressing shRNA-Yap. In A-D, representative confocal images were shown in (A, C), Scale bar 10µm; and quantification data were presented in (B, D). (Mean ± SD, n =20 from 3-different cultures). *, P < 0.05.

**Supplemental Fig. 9.**


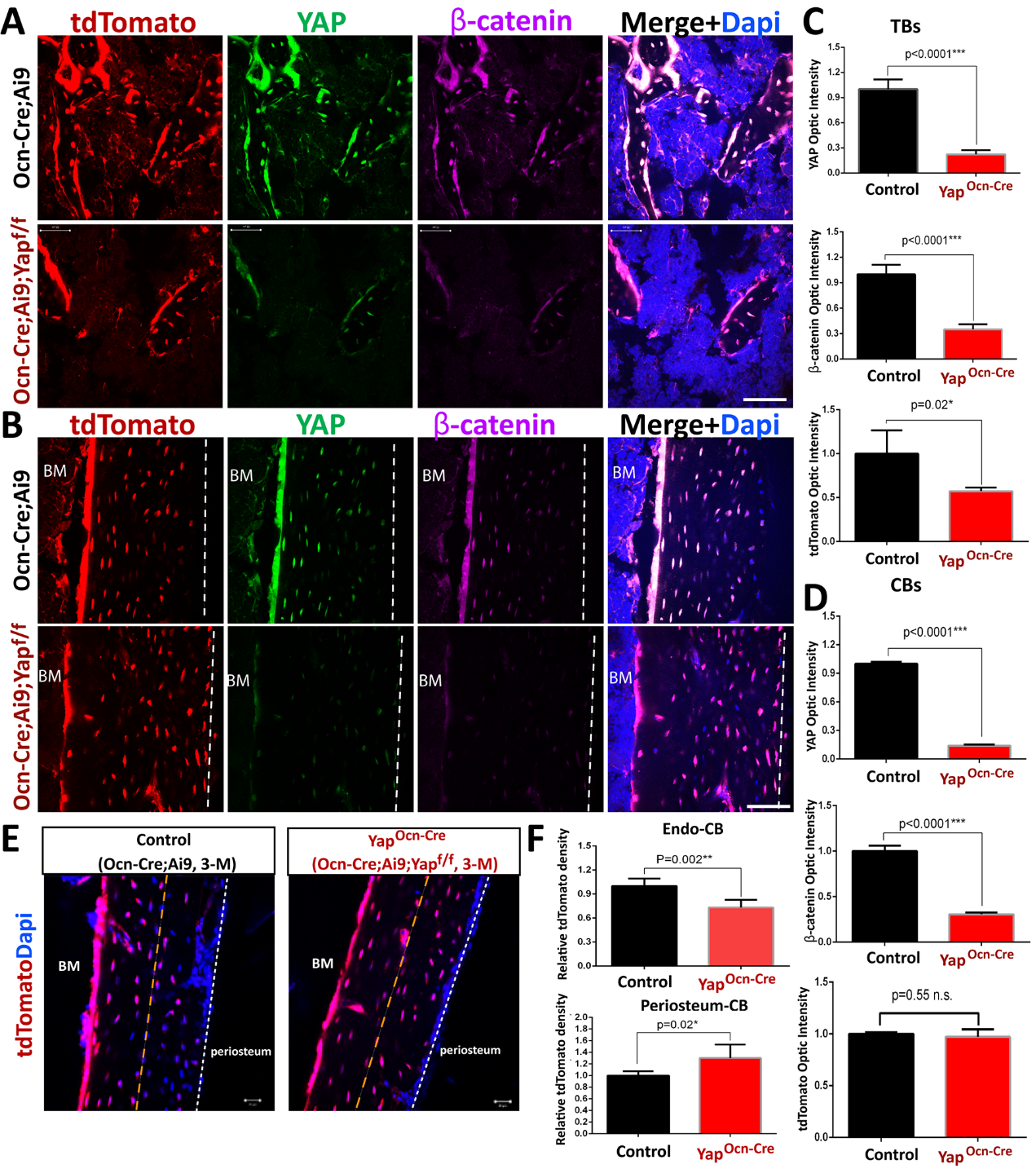


**Supplemental Fig. 9. Reductions in β-catenin levels and tdTomato positive OB-lineage cells in Yap^Ocn-Cre^ femurs.** Femurs from Ocn-Cre; Ai9 (control) and Ocn-Cre; Ai9; Yap^f/f^ mice (3-M old) were subjected to immunohistochemical staining analyses using indicated antibodies (YAP/TAZ, #8418, CST; β-catenin, C7207, Sigma). Representative co-focal images were shown in (**A-B, E**) (midshaft of CB in E); Scale bar, 50µm; and the quantification data were presented in (**C, D, and F**). (Mean ± SD, n =3 from 3-different male mice).

**Supplemental Fig. 10.**


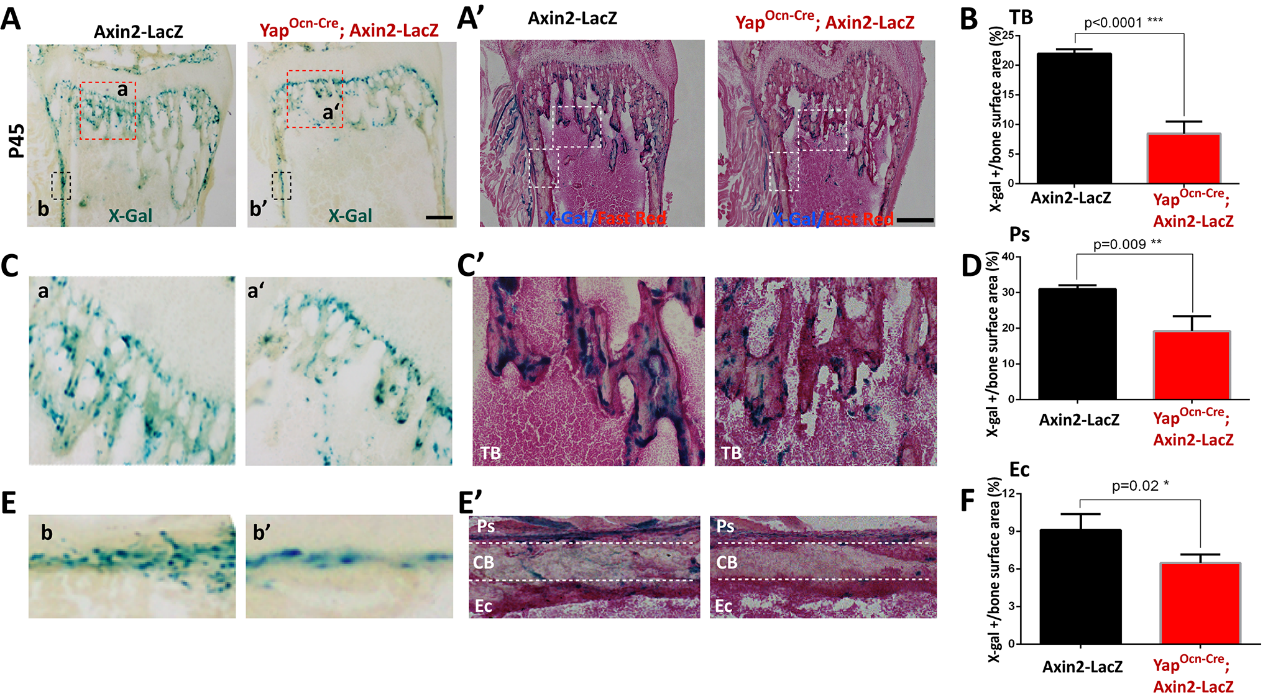


**Supplemental Fig. 10.** **β-catenin signaling reporter (LacZ) analysis.** Femur sections from control (Axin2-LacZ) and Yap^Ocn-Cre^; Axin2-LacZ mice at P45 were stained for their X-gal enzymatic activity. In (**A, C, and E**), X-gal staining images without counterstaining; and in (A’, C’, and E’), X-gal staining was counter-stained with Fast Red. Images in (C, E) and (C’, E’) were amplified images from marked squares in (A) and (A’), respectively. Scale bars, 200µm. Quantification analyses were presented in (**B, D, and F**). The values of mean ± SD from 3 different mice were presented.

**Supplemental Fig. 11.**


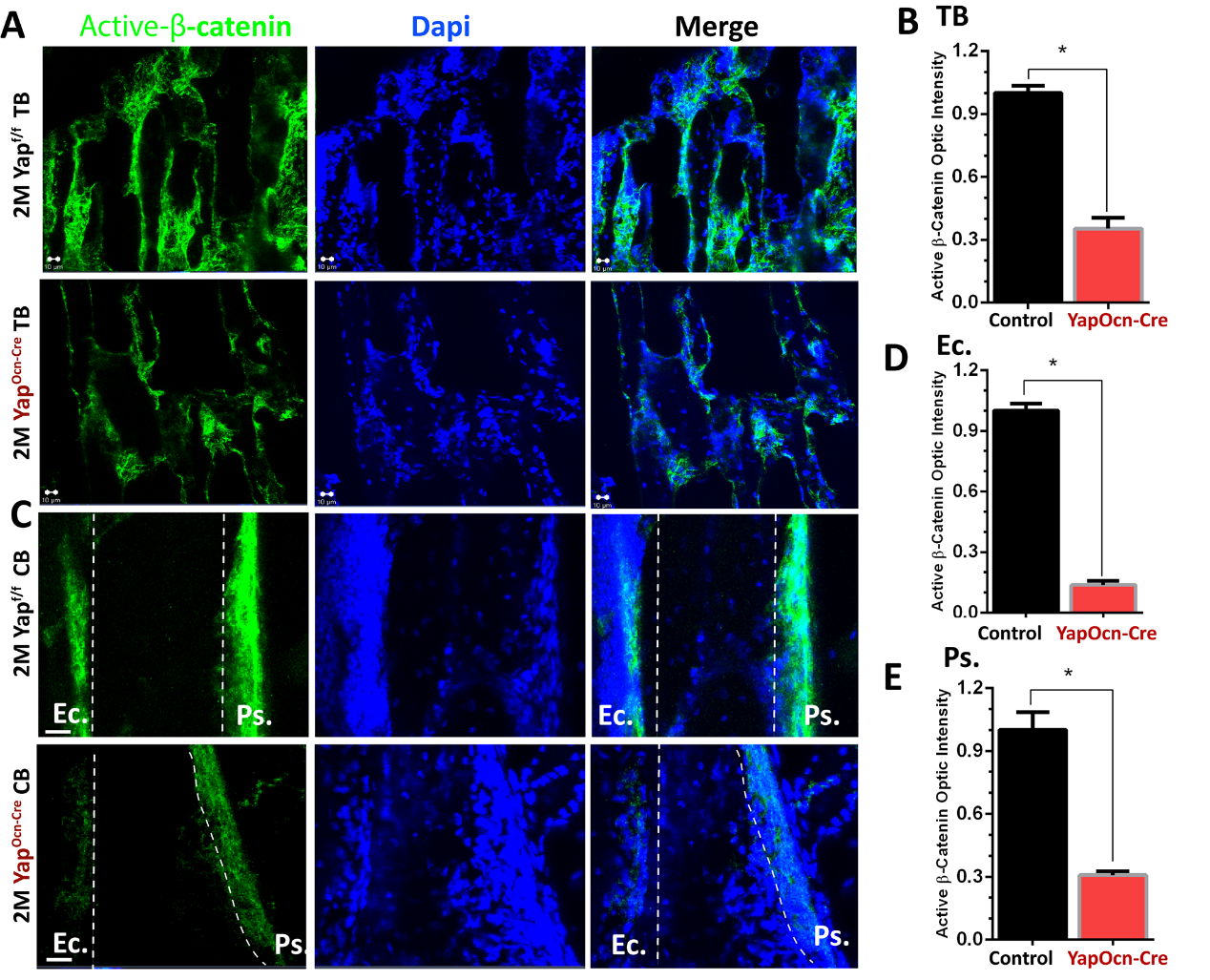


**Supplemental Fig. 11. Reductions in active β-catenin levels in Yap^Ocn-Cre^ femurs.** Femurs from Yap^f/f^ (control) and Yap^Ocn-Cre^ mice (2-M old) were subjected to immunostaining analyses using indicated antibodies (#8814, CST). Representative co-focal images were shown in (**A, C**) (midshaft of CB in C); Scale bar, 10µm; and the quantification data were presented in (**B, D, and E**). (Mean ± SD, n =3 from 3-different male mice).

**Supplemental Fig. 12.**

**
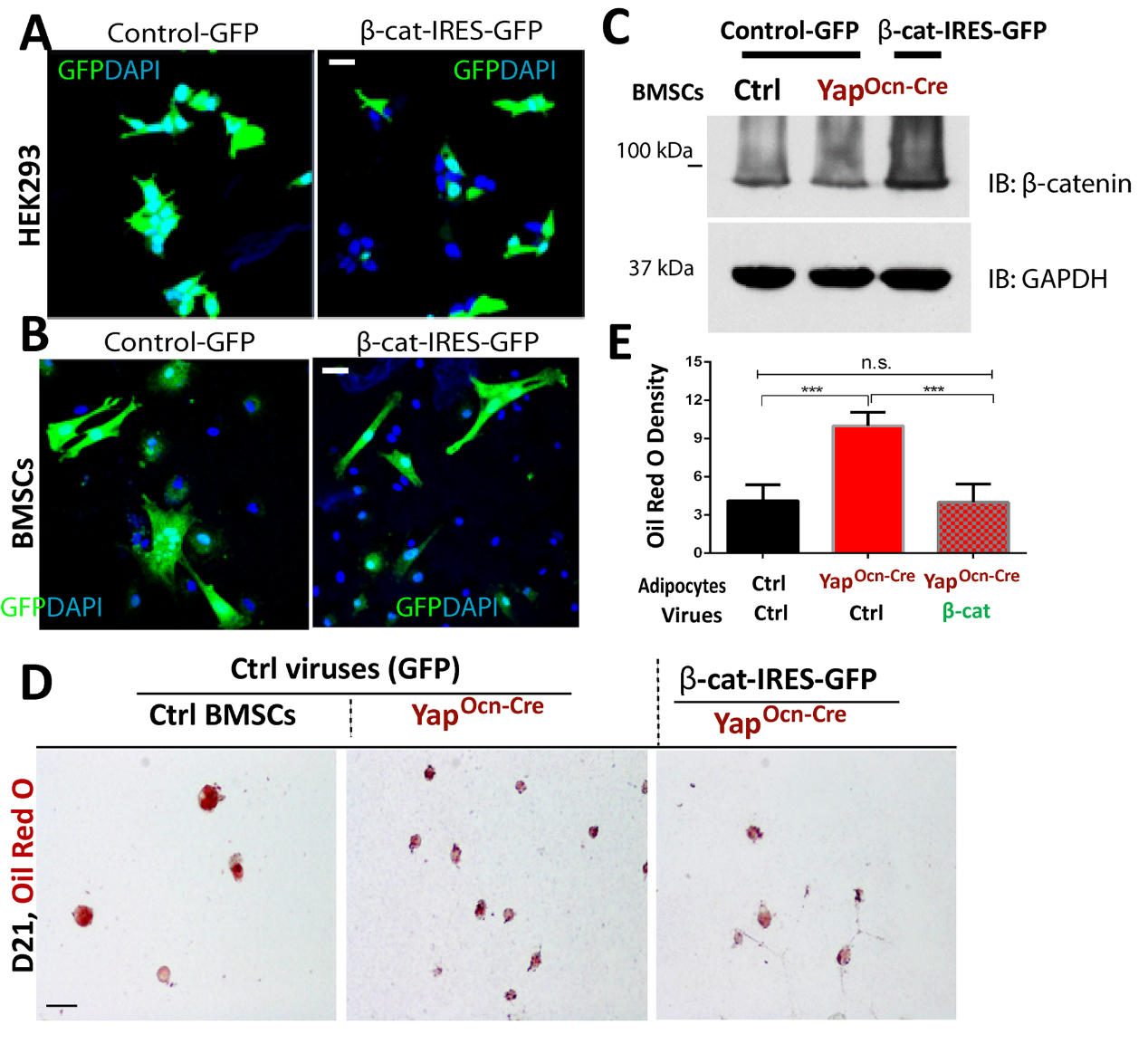
Supplemental Fig. 12.** **Retroviruses encoding GFP (control) or β-catenin-IRES-GFP and rescued adipocytes over-formation by expression of β-catenin in Yap^Ocn-Cre^-BMSCs.** The retroviruses were generated by transfection of the plasmids into 293GPG packing cell line, and used for infection in 293T cells **(A)** and BMSCs **(B)**, Scale bar 20µm. Cell lysates of infected BMSCs were subjected to Western blot analysis using indicated antibodies **(C)**. **(D-E)** Control (Ctrl) BMSCs and Yap^Ocn-Cre^ BMSCs (isolated from 3-M old mice) were infected with ctrl retroviruses (GFP) and retroviruses encoding β-catenin-IRES-GFP. BMSCs were then subjected to adipocyte differentiation assay. At indicated date of cultures, cells were stained for Oil Red O. Representative images were shown in (D), Scale bar 100µm. Quantification data were presented in (E). (Mean ± SD, n =50 from 3-different cultures). *, P < 0.05.

**SUPPLEMENTAL METHODS**

**In vitro BMSC, OB, and adipocyte cultures**

BMSC culture was carried out by use of a standard protocol as described previously ^(1, 2, 3, 4)^. In brief, the whole bone marrow cells flushed out from long bones of mice with DMEM were filtered through a 70-mm filter mesh, washed, re-suspended, and then plated in 100-mm dishes with growth medium (DMEM plus 10% FBS), which were incubated at 37^o^C with 5% CO2. The non-adherent cells were removed 72 hours after medium changing. The attached BM cells were cultured with the growth medium for 7 days. These cells were then resuspended and plated at plates or culture dishes, and cultured for another 3-6 days with the same growth medium. These cells, so called BMSCs, were used for Western blot, immune-staining analysis, or subjected to further differentiation with OB or adipocyte differentiation medium.

OBs and adipocytes were in vitro differentiated from BMSCs as described previously^(3)^. To induce OB differentiation, BMSCs, plated at densities of 1*10^4^/cm^2^ at 12-well plates, were cultured in osteogenic medium (DMEM containing 10% FBS, 1%P/S, 10mM β-glycerophosphate and 50μM L-Ascorbic Acid-2- phosphate). At day (D) 14 cultures, cells were subjected to ALP (alkyline phosphatase) staining and quantification analyses as described previously ^(1, 2, 3, 5)^. At D14, cells were also used for Western blot, immune-staining or RT-PCR analyses. At D21, cells were washed with PBS, fixed with 4% paraformaldehyde for 10 minutes, and incubated with 40mM alizarin red S solution (Sigma) for 30 min at RT according to the manufacturer’s protocol. To induce adipocyte formation, BMSCs, plated at 3*10^4^/cm^2^ at 12-well plates, were exposed to adipocyte differentiation media (DMEM containing 10% FBS, 1% P/S, 0.5 mM isobutylmethylxanthine, 10 µg/ml insulin and 1*10^-6^M dexamethasone) for 3-Ds, followed by a 9-D incubation with maintenance media (growth medium plus 10 µg/ml insulin), which were replaced every other day. The cells were monitored daily under a microscope for the appearance of lipid droplets, which were confirmed by Oil-Red O staining as described previously ^(3)^. Oil Red O solution was freshly prepared by diluting a stock solution (0.5 g of Oil Red O in 100 ml of isopropanol) with water (6:4) followed by filtration.

**CFU-F, CFU-OB, CFU-AD assays**

For CFU-fibroblast(CFU-F) assay, bone marrow cells (BMCs) were flushed out from mouse femurs and tibiae and seeded in triplicate cultures (6-well plates) at 1.0 × 10^6^ cells/well, and cultured using complete media (MesenCult™ Basal Medium and Mesenchymal stem cell Stimulatory Supplements) (StemCell Technologies; Vancouver, BC, Canada). 24 hours after seeding, the media that contained non-adherent cells were removed and replaced with new complete media. The attached cells were cultured continuously in the complete media with fresh media replaced every third day. The formation of CFU-F was evaluated after 14-D culture by Crystal Violet Staining, and the colonies (>50 cells) were counted using light microscopy.

For the CFU-osteoblast (CFU-OB) assay, 1× 10^5^ nucleated cells were seeded in each well of 12-well plates in triplicate. 24 hours later, the growth media were removed from the cells and replaced with osteogenic media (complete media plus 50 μM ascorbic acid-2-phosphates, 10 mM β-glycerophosphate), and cultured in osteogenic media continuously for 21 days with fresh media replaced every third day. The cells were washed with PBS twice, fixed with 4%PFA, and stained with Alizarin Red S Staining (Sigma-Aldrich), and the colonies were scored.

For the CFU-adipocyte (CFU-AD) assay, 1 × 10^6^ nucleated cells were seeded in each well of 12-well plates in triplicate and treated with adipocyte differentiation media (complete media with 0.5 mM isobutylmethylxanthine, 10 µg/ml insulin and 1*10^-6^M dexamethasone) for 3 days, followed by a 9-day incubation with maintenance media (complete medium plus 10 µg/ml insulin), which were replaced every other day. The cells were monitored daily using a microscope for the appearance of lipid droplets. For Oil-Red O staining, the cells were washed with PBS, fixed in 4%PFA for 15min, and stained with fresh Oil Red O solution, and the colonies were scored.

**In vitro BMM culture, OC-differentiation by RANKL, and OC-differentiation assay by co-culture of BMMs with OBs**

Mouse BMMs were generated as described previously ^(1, 2, 3, 6)^. In brief, the bone marrow was flushed from femurs and tibiae of 2-3 month old Yap^f/f^ and Yap^Ocn^-CKO mice with ice-cold α-MEM and plated on 100 mm tissue culture plates in α-MEM containing 10% FBS and 10 ng/ml recombinant M-CSF (macrophage colony-stimulating factor). Cells were incubated at 37°C with 5% CO_2_ overnight. Non-adherent cells were harvested and subjected to Ficoll-Hypaque gradient centrifugation for purification of BMMs.

Mouse OC-genesis by RANKL was carryout as described previously ^(1, 2, 3, 6)^. In brief, 5×10^4^ BMMs were incubated with OC differentiation medium containing 10 ng/ml recombinant M-CSF and 100 ng/ml recombinant RANKL (receptor activator of NFκB ligand). OCs (multi-nucleated, large spread cells) began to form at D5 after RANKL treatment. The cells were then subjected to TRAP (tartrate-resistant acid phosphatase) staining to confirm their OC identity.

Mouse OC-genesis by co-culture of BMMs with OBs was carried out as described previously ^(7)^. In brief, BMMs from WT mice (3-M old) (at a density of 1 × 10^4^ per well) were mixed with OBs isolated from long bones of 3-M old control and Yap^Ocn-Cre^ mice. These cells were co-cultured with αMEM supplemented with 10% FBS and 10^−8^ M 1, 25 dihydroxyvitamin D for 10-Ds, and then subjected to TRAP staining analysis.

**Western blot analysis**

Cultured cells were lysed in the lysis buffer (50 mM Tris-HCl (pH 7.4), 150 mM NaCl, 1% NP-40, 0.5% Triton X-100, 1 mM phenylmethylsulfonyl fluoride (PMSF), 1 mM EDTA, 5 mM sodium fluoride, 2 mM sodium orthovanadate and protease inhibitor cocktail) for 30 min on ice and centrifuged at 12,000 rmp for 20 min, and protein concentration was determined by BCA protein assay kit (Thermofisher). Proteins were separated by 8%-12% SDS-PAGE gel electrophoresis and transferred onto the nitrocellulose (NC) membrane. Blotted membranes were blocked in 10% skim milk at room temperature for 1-hr and incubated with primary antibody overnight at 4°C, rinsed and incubated for 1-hr at room temperature with an appropriate horseradish-peroxidase-conjugated secondary antibody (1:5,000, Invitrogen). Chemiluminescent detection was performed with the ECL kit (Pierce, Rockford, IL). Primary antibodies included mouse monoclonal anti-YAP (1:1,000, WH0010413M1, Sigma), anit-p-Smad1/5/8 (1:1,000, #13820, CST), anti-β-catenin (1:2000, two different antibodies: C7207 and C2206 Sigma), anti-Active-β-catenin (1:1000, #8814, CST), anti-TAZ(1:1000, T4077,CST) and anti-DKK1(1:1000, MAB1765), anti-β-actin or GAPDH as a loading control was detected alongside the experimental samples (Sigma). For chemiluminescent signals analysis, protein bands detected by ECL were scanned into pictures and analyzed using Image J software.

**Micro-computed tomography (µCT)**

Micro-architecture of the distal trabecular bone and midshaft cortical bone of the femur were measured by Scanco µCT 40 (Scanco Medical AG, Brüttisellen, Switzerland) as described previously ^(1, 2, 3, 5)^ . The 3-D reconstruction of the trabecular bone was performed using all the outlined slices. No cortical bone was included in this analysis. Trabecular parameters were assessed at the distal femoral metaphysis for trabecular parameters, including the trabecular bone volume fraction (BV/TV), trabecular bone thickness (TB.Th), trabecular number (TB.N) and trabecular separation (TB.Sp).The scan of the cortical bone was performed at the midshaft of the femur and consisted of 25 slices (each slice was 12µm in thickness). Scans were reconstructed as for the trabecular scans and the region of interest was drawn on all 25 slices. There was no trabecular bone in these images at the midshaft. Cortical bone was thresholded at 329, and the 3-D reconstruction was performed on all 25 slices. Data was obtained on BV, TV, BV/TV, cortical thickness (CB.Th), periosteal perimeter (Ps. Perimeter), endosteal perimeter (Ec. Perimeter), cortical Cross-section area and mean polar moment of inertia (MMI).

**Bone histomorphometric analysis**

Bone histomorphometric analyses were carried out as previously described^(5)^. In brief, mouse tibia and femurs were fixed overnight in 10% buffered formalin, decalcified in 14% EDTA, embedded in paraffin, sectioned, and subjected for H&E, TRAP, and Goldner’s Trichrome stain analyses. Bone histomorphometric perimeters were determined by measuring the areas situated at least 0.5 mm from the growth plate, excluding the primary spongiosa and trabeculae connected to the cortical bone.

**Bone dynamic histomorphometric analysis**

To obtain the bone formation rate, P16/P76 mice were injected with the fluorochrome labels calcein green (10 mg/kg, Sigma–Aldrich) intraperitoneally, and followed by another injection 12 days later. Mice were sacrificed two days after the second injection (P30/P90). Mouse tibia and femurs were fixed overnight in 70% ethanol. Images were obtained using a 25× objective (LSM510; Carl Zeiss).

**Measurement of serum levels of osteocalcin and PYD**

Mouse blood was obtained by cardiac puncture. Samples were allowed to clot for at least 30 minutes and then centrifuged for 10 minutes at 3000 rpm. Serum was collected and frozen at −80 °C until use. Mouse serum levels of osteocalcin were measured by use of mouse osteocalcin Elisa kit (QUIDEL Corporation). The serum levels of PYD were determined by use of METRA Serum PYD EIA kit (QUIDEL Corporation). All the assays were carried out per the instructions. All the ODs measured after reactions were converted to osteocalcin/PYD concentration using their standard curves. All the samples were measured in duplicate, and values were subjected to statistical analysis.

**MC3T3 cell culture, transfection of shRNA-Yap, and generation of Yap-KO MC3T3 cell line**

MC3T3-E1 cells were grown in DMEM containing 10% (vol/vol) FBS, and 50 units/ml penicillin and streptomycin. Cells plated at 1*10^4^/well onto 12-wells coverslips the day before transfection. Cells were transfected with Control-GFP Vector or shRNA-Yap-GFP by Lipofectamine 3000 (Invitrogen).

The Yap shRNA was designed by web-based Invitrogen BLOCK-iT RNAi designer. The target sequence as follow: 5’-GGAAGCGCTGAGTTCCGAAAT-3’ was subcloned to pLL3.7 vector (Addgene) with following conditions: vector (50 ng), T4 ligase (NEB, 1 µl), shRNA Oligo (7 µl), 10x T4 ligation buffer (NEB, 1 µl) at 16^o^C overnight. 96h after transfection, cells were fixed with 4% paraformaldehyde at room temperature for 20 min, permeabilized in 0.05% Triton X-100 for 10 min, and then subjected to co-immunostaining analysis using anti-Yap and anti-β-catenin antibodies.

For generation of Yap-KO MC3T3 cell line, PX330 vector was purchased from Addgene. The puromycin resistant gene was replaced by GFP. Guidance RNA GCCGCCGTCCGTGTCTCCGGC for Yap was inserted into Bbsl restriction site and verified by sequencing. The PX330 plasmid was then transfected to MC3T3 cells and GFP positive cells were sorted by fluorescence-associated cell sorting(FACS) and cultured in 96-well plate with one cell per well. 4-5 days later, the cells were passed to 12-well plate, and the Yap-KO clones were identified by western blot and PCR-based genomic DNA sequencing.

**Immunostaining analysis**

Mouse femurs and tibia were fixed in fresh 4% paraformaldehyde (PFA) for one day, decalcified in 14% EDTA, cryopreserved in O.C.T. compound for bone sectioning. Longitudinal sections of 20μm were cut on a freezing microtome and then immediately processed for immunostaining analysis. For cultured BMSCs and OBs (BMSCs were obtained by culturing attached BM cells from mouse femur bones for 3-6-days with growth medium. OBs are in vitro differentiated from BMSCs with OB differentiation medium for another 14 days), the cells were fixed with fresh 4% PFA in 0.1 M PBS (pH 7.4) for 15 min. The analysis was performed using a blocking solution containing 10% goat serum and 0.05% Triton X-100 in 0.1M PBS. The primary antibodies used were rabbit polyclonal antibodies against β-catenin (1:2000, C2206, Sigma), anti-Ki67 (1:200, AB9260, Millipore), anti-YAP(1:200, #4912, CST), anti-YAP/TAZ (1:200, #8418/D24E4, CST) or with a monoclonal antibodies against-YAP (1:200, WH0010413M1, Sigma) or anti-β-catenin (1:2000, C7207, Sigma), rat polyclonal antibodies against anti-BrdU (1:1000, Accurate Chemical & Scientific Corporation), and anti-Active-β-catenin (1:1000, #8814, CST). Bone sections or cells were co-stained for DAPI (1: 1,000, Sigma) to visualize nucleus. No positive signal was observed in control incubations using no primary antibody. Images were acquired on a Zeiss confocal system (FM300) using a multi-track configuration and processed using Zeiss confocal software and Adobe Photoshop CS 6.0 software.

**Retrovirus generation and infection**

The 293GPG packaging cell line ^(8)^ was maintained in 293GPG medium (Dulbecco’s Modified Eagles Medium (DMEM) with high glucose, L-glutamine and sodium pyruvate supplemented with 10% heat-inactivated FBS, 1ug/ml tetracycline, 2ug/ml puromycin, and 300ug/ml G418) as previously described ^(8)^. a 90% confluent 100mm plate of 293-GPG cells was spited into 4 new plates containing 10 ml fresh in DMEM+10% FBS (+1ug/ml tetracycline, 2ug/ml puromycin, and 300ug/ml G418)] for each retroviral construct. To produce virus, 293GPG cells culture media was first replaced with DMEM supplemented with 10% heat-inactivated FBS and penicillin/ streptomycin, free of tetracycline, puromycin and G418. Cells were then transiently transfected with 15μg of plasmid DNA using Lipofectamine 3000 (Invitrogen). In this study, we used either the MSCV-beta-catenin-IRES-GFP retroviral vector (addgene plasmid#14717) ^(9)^ or Control-GFP vector. The medium was changed every 24 hours. Viral supernatant was collected at 72, 96, 120, 144, and 168 hrs. After spinning cells out of media, the supernatant was divided into 1 ml aliquots (in sterile tubes) and frozen at –80 degree until use.

To infect BMSCs, BMSCs were plated in densities of 1*10^4^/cm^2^ and 3*10^4^/cm^2^ for OB and adipocyte inductions, respectively. In the following day, cells were replaced their medium with 1 ml of virus + 1 ml of fresh complete media + polybrene (1/1000, sigma H-9268). 3 days later, the culture media were replaced with OB differentiation media and adipocyte differentiation media, respectively.

**Flow cytometry analysis**

For bone marrow cells (BMCs), bone marrow was flushed from femurs and tibias of 3-M old Ocn-Cre;Ai9 and Ocn-Cre;Ai9;Yap^f/f^ mice, and red blood cells were lysed with ACK Lysing buffer (Thermofisher [A1049201](https://www.thermofisher.com/order/catalog/product/A1049201)). The pellet cells were collected by centrifugation at 300 x g for 5 minutes at room temperature. After gently mixing the pellet cells with 2 ml cold phosphate buffered saline (PBS), cells were re-centrifugated at 300 x g for 5 minutes at 2-8°C. The pellet cells were resuspended in 1 ml PBS with 1% FBS, which were subjected for flow cytometry analysis.

For BMSCs, the attached BM cells were cultured with the growth medium for 7 days. These cells were then resuspended and plated at 100mm culture dishes, and cultured for another 3 days with the same growth medium. Then cell media were removed from culture dishes and cells were rinsed with PBS. Trypsin solution was added to incubate at 37°C for 2 min. the detached adherent cells were centrifuged and the pellet cells were washed with 1 ml cold PBS, and finally resuspended in 0.5 ml PBS with 1% FBS for flow cytometry analysis. Flow cytometric analysis was performed by use of a flow cytometer (FACSCanto; BD) in AU core facility. Acquisition and analysis were performed by using FACSDiva 8.0.1 software (BD).

**RNA isolation and Real-Time PCR**

Total RNAs were isolated from primary cultured BMSCs, OBs (D14 culture), ADs (adipocytes, D21 culture), or MC3T3 by Trizol extraction (Invitrogen, Carlsbad, CA, USA). Quantitative PCR was performed with Quantitect SYBR Green PCR Kit (Qiagen) according to the manufacturers’ instructions and a Real-Time PCR System with analytical software (Opticon Monitor 3). The following primers were used: YAP, 5′-AGGAGAGACTGCGGTTGAAA-3′ and 5′-CCCAGGAGAAGACACTGCAT-3′; β-catenin, 5′-GAATGAAGGCGTGGCAACA-3′ and 5′-CAGTCCAAGATCTGCAGTCTCATT-3′; Cyclin D1, 5′-CGCCCTCCGTATCTTACTTCAA-3′ and 5′-TCTTCGCACTTCTGCTCCTCAC-3′; RUNX2, 5′-TGACATCCCCATCCATCCAC- and –AGAAGTCAGAGGTGGCAGTG-3′; Osterix, 5′-GGGGAAAGGAGGCACAAAGA-3′ and 5′-AGGAAATGAGTGAGGGAAGGGT-3′; COL1A1, 5′-CCTACTCAGCCGTCTGTGCCT-3′ and 5′-GCCCTCGCTTCCGTACTCG-3′; Osteocalcin, 5′-CTGACCTCACAGATGCCAA-3′ and 5′-GGTCTGATAGTCTGTCACAA-3′; RANKL, 5′-ATCCCATCGGGTTCCCATAA-3′ and 5′-TCCGTTGCTTAACGTCATGTTAG-3′; OPG, 5′-GGCCTGATGTATGCCCTCAA-3′ and 5′-GTGCAGGAACCTCATGGTCTTC-3′; DKK1, F: 5'-TACCAGACTCTTGACAACTACCAGC-3' R: 5'-TTTCGGCAAGCCAGACAGAT-3'; PPAR-γ, 5′-TATCACTGGAGATCTCCGCCAACAGC-3′ and 5′-GTCACGTTCTGACAGGACTGTGTGAC-3′; FABP4, 5′-TGAAAGAAGTGGGAGTGGGCT-3′ and 5′-TCCTGTCGTCTGCGGTGATT-3′; Adipsin, 5′-GCTATCCCAGAATGCCTCGTT-3′ and 5′-GGTTCCACTTCTTTGTCCTCGTAT-3′; LPL, 5′-ACTGAGGATGGCAAGCAACACA-3′ and 5′-ATGAGCAGTTCTCCGATGTCCA-3′; GAPDH primers (5′- AAGGTCATCCCAGAGCTGAA -3′ and 5′-CTGCTTCACCACCTTCTTGA-3′ were used for normalization.

**Supplemental references**

1. Xiong L, et al. Lrp4 in osteoblasts suppresses bone formation and promotes osteoclastogenesis and bone resorption. Proc Natl Acad Sci U S A. 2015;112(11):3487-92.

2. Cui S, et al. APPswe/Abeta regulation of osteoclast activation and RAGE expression in an age-dependent manner. J Bone Miner Res. 2011;26(5):1084-98.

3. Xia WF, et al. Swedish mutant APP suppresses osteoblast differentiation and causes osteoporotic deficit, which are ameliorated by N-acetyl-L-cysteine. J Bone Miner Res. 2013;28(10):2122-35.

4. Nadri S, et al. An efficient method for isolation of murine bone marrow mesenchymal stem cells. Int J Dev Biol. 2007;51(8):723-9.

5. Zhou Z, et al. Regulation of osteoclast function and bone mass by RAGE. J Exp Med. 2006;203(4):1067-80.

6. Zhou Z, et al. HMGB1 regulates RANKL-induced osteoclastogenesis in a manner dependent on RAGE. J Bone Miner Res. 2008;23(7):1084-96.

7. Iezaki T, et al. The Transcriptional Modulator Interferon-Related Developmental Regulator 1 in Osteoblasts Suppresses Bone Formation and Promotes Bone Resorption. J Bone Miner Res. 2016;31(3):573-84.

8. Ory DS, Neugeboren BA, Mulligan RC. A stable human-derived packaging cell line for production of high titer retrovirus/vesicular stomatitis virus G pseudotypes. Proc Natl Acad Sci U S A. 1996;93(21):11400-6.

9. Reya T, et al. A role for Wnt signalling in self-renewal of haematopoietic stem cells. Nature. 2003;423(6938):409-14.
